# Development of soil surface wetness models using machine learning techniques in the selected sites in Punjab, North-Western India

**DOI:** 10.1038/s41598-026-50687-9

**Published:** 2026-05-26

**Authors:** Sushindra Kumar Gupta, Bibhuti Bhusan Sahoo, P. K. Singh, Ozgur Kisi, P. C. Nayak, L. N. Thakural, Sumant Kumar, Christoph Külls

**Affiliations:** 1 Groundwater Hydrology Division, National Institute of Hydrology, Roorkee, 247667 India; 2 Department of Agricultural Engineering, Centurion University of Technology and Management, Paralakhemundi, Odisha 761211 India; 3 Department of Soil and Water Conservation Engineering, Sardar Vallabhbhai Patel University of Agriculture and Technology, Meerut, 761211 India; 4 Department of Civil Engineering, Technical University of Applied Sciences, 23562 Lübeck, Germany; 5 Department of Civil Engineering, School of Technology, IIia State University, 0179 Tbilisi, Georgia; 6 School of Civil, Environmental and Architectural Engineering, Korea University, Seoul, 02841 South Korea; 7 Surface Water Hydrology Division, National Institute of Hydrology, Roorkee, 247667 India

**Keywords:** Machine learning, Bagging, AdaBoost, Soil surface wetness, Punjab, Climate sciences, Engineering, Environmental sciences, Hydrology, Natural hazards

## Abstract

Accurate prediction of soil surface wetness (SSW) is vital for effective land management and resource optimization, particularly in sensitive ecosystems like the Western Himalayas. The main objective of the present study is to improve the accuracy of SSW prediction using hybrid and bagging models in the selected sites in Punjab, north-western India. The study utilizes ten machine-learning models comprising five base learners-random forest (RF), extreme gradient boosting (XGBoost), light gradient boosting machine (LightGBM), logistic model tree (LMT), and classification and regression tree (CART) and their corresponding AdaBoost-based hybrid variants: AdaBoost-RF, AdaBoost-XGBoost, AdaBoost-LightGBM, AdaBoost-LMT, and AdaBoost-CART. The SSW data set was collected from NASA POWER platform and Goddard Earth Observing System (GEOS) derived data which covers the period from 1986 to 2021. For model development, we applied a monthly data-lagging technique to generate different model scenarios. For feature selection, we applied greedy stepwise and best-first algorithms to identify the most effective predictors and improve model efficiency. Evaluations of the models were based on a variety of statistical indices. The results show that the RF model achieved the highest correlation coefficients (CC) across the study areas, ranging from 0.64 to 0.76 in Moga, 0.61 to 0.82 (XGBoost) in Hoshiarpur, and 0.41 to 0.64 (LMT) in Firozpur during the testing period. Accordingly, the hybrid AdaBoost-LightGBM model was the best, with CC values of 0.61–0.76, 0.63–0.82, and 0.49–0.60 for Moga, Hoshiarpur, and Firozpur, respectively. Overall model performance was limited to moderate and varied across locations and scenarios; although AdaBoost–LMT showed the best relative performance, the results primarily support its use for reproducing temporal variability in GEOS-derived SSW rather than precise wetness estimation. The findings contribute to improving SSW estimation and support data-driven decision-making for sustainable land and water management in the selected sites in Punjab, north-western India.

## Introduction

Soil surface wetness (SSW) plays a critical role in understanding hydrological, agricultural, and ecological processes, particularly in sensitive and complex terrains such as the Western Himalayas. In this region, characterized by diverse climatic conditions and complex topography, SSW significantly influences water availability, vegetation patterns, and soil health. Accurately estimating SSW is essential for applications such as water resource management, agricultural planning, and climate impact studies^1,2^. Also, for slope stability and land-slide risk assessment SSW plays a critical role. However, traditional methods of estimating soil wetness, which rely on field measurements and empirical models, are often constrained by their high cost, labour-intensive nature, and limited ability to capture spatial heterogeneity effectively^3^.

Understanding and forecasting SSW is of paramount importance for sustainable agricultural practices. Empirical models based on statistical correlations between soil moisture and meteorological variables have been frequently used in early research^4–7^. Although these models offered valuable insights, they often struggled to capture the complexity of non-linear relationships in regions with diverse environmental conditions^8,9^. Using mathematical depictions of hydrological processes, physically-based models like the soil water assessment tool (SWAT) aim to simulate soil moisture dynamics^10,11^. Despite providing more detailed mechanistic insight, these models are computationally demanding and often fail to adequately capture regional variations in soil properties. Remote sensing and GIS-based approaches have become increasingly common for soil moisture estimation^12,13^.

Recent advances in soil science increasingly recognize soil moisture and soil surface wetness (SSW) as central variables linking hydrological processes with soil erosion, soil organic carbon (SOC) dynamics, vegetation restoration, and climate adaptation^14,15^. Beyond controlling infiltration and evapotranspiration, SSW regulates runoff generation, hydrological connectivity, and the susceptibility of soil to erosion, particularly under changing land-use and management conditions^14^. Contemporary studies in soil conservation and ecosystem research have demonstrated that moisture-driven connectivity and threshold responses strongly influence erosion and SOC losses on restored and managed landscapes^14,15^. In this broader context, machine learning (ML) approaches are increasingly employed not only for moisture prediction but also for identifying nonlinear interactions among soil, vegetation, and climate drivers^14–16^. Consequently, developing robust ML-based SSW models has relevance that extends beyond hydrological forecasting to support soil conservation planning, land management, and climate-resilient agriculture, thereby motivating the present focus on flexible, nonlinear ensemble and hybrid ML frameworks.

Recent advances in machine learning (ML) have enabled innovative tools to improve prediction accuracy and reliability. Hybrid ML techniques, which combine the strengths of multiple models, offer promising solutions for addressing the complexities of SSW prediction. This study investigates a hybrid modeling framework incorporating ten machine-learning models-RF, XGBoost, LightGBM, LMT, CART, AdaBoost-RF, AdaBoost-XGBoost, AdaBoost-LightGBM, AdaBoost-LMT, and AdaBoost-CART to enhance the accuracy of SSW estimation. within a hybrid modeling framework to enhance SSW estimation accuracy^17–19^^20^;. By leveraging the complementary strengths of these models, the research aims to provide a robust methodology for precise and reliable SSW predictions in the selected sites in Punjab, north-western India.

This study aims to improve the understanding of SSW prediction challenges in the selected sites in Punjab, north-western India, contributing to agricultural and environmental sciences. Monitoring and understanding surface soil wetness is critical for efficient water resource management, precision agriculture, and reducing the risks of droughts and floods^21–24^. The machine learning models developed in this study were trained and tested using NASA Goddard Earth Observing System (GEOS)-based modeled SSW outputs as reference data. The objective of the modeling framework is therefore to construct surrogate predictive models that reproduce the statistical patterns and temporal variability of climate-model-derived SSW, rather than to validate against in-situ soil moisture measurements. This methodological choice reflects the practical constraints of limited long-term ground observations in the study region and aligns with previous studies employing reanalysis-based datasets for hydrological and land surface modeling applications. Research in this area employs advanced methodologies, including satellite observations, sensor networks, and computational modeling, to assess soil moisture dynamics and their impact on ecosystems.

While previous studies have made valuable contributions, a notable gap exists in the exploration of machine-learning models specifically tailored to the selected sites in Punjab, north-western India^25–28^. The performance of RF, XGBoost, LightGBM, LMT, CART, AdaBoost-RF, AdaBoost-XGBoost, AdaBoost-LightGBM, AdaBoost-LMT, and AdaBoost-CART for this application has not yet been sufficiently explored. The need for more accurate and interpretable models that can handle the region’s complex topography, diverse soil types, and variable climatic conditions underscores the importance of the present study^14,29,30^. By integrating machine learning techniques, this research aims to develop a more robust approach to predicting soil surface wetness in these challenging geographical and climatic settings. In addition, several widely used hybrid and ensemble methods such as random forest, gradient boosting machines (GBM), Extreme Gradient Boosting (XGBoost), and AdaBoost employ CART as their core base learner. In these approaches, multiple CART-based trees are combined to improve predictive performance, robustness, and generalization. CART is not only used as a standalone Decision Tree algorithm but also serves as the underlying structure in many hybrid and ensemble learning frameworks applied in hydrology and groundwater modeling.

Among ensemble learning strategies, boosting-based approaches-particularly AdaBoost-have received increasing attention for their ability to enhance the predictive capability of relatively simple base learners by iteratively focusing on difficult-to-predict instances and reducing systematic bias. When combined with tree-based or semi-parametric learners, AdaBoost can offer improved robustness in the presence of nonlinear relationships and noisy target signals. Given these properties, AdaBoost-based hybrid models are especially relevant for emulating complex, model-derived soil surface wetness products, where temporal persistence and regime-dependent behaviour vary across locations. Accordingly, the present study places particular emphasis on evaluating AdaBoost-hybrid formulations in comparison with bagging-based models to assess their relative effectiveness as surrogate predictors of NASA POWER (GEOS-derived) SSW.

Despite the growing use of machine learning in soil moisture and wetness modeling, most studies either predict observed soil moisture using exogenous hydro-meteorological drivers or apply increasingly complex temporal architectures for sequence learning. In contrast, the present study addresses a narrower but practically relevant question: whether monthly NASA POWER (GEOS-derived) soil surface wetness can be emulated by interpretable, computationally efficient tree-based ensemble and hybrid models using only lagged SSW inputs. The contribution of this work, therefore, does not lie in claiming broad algorithmic novelty or superiority over the full range of modern time-series approaches, such as recurrent or deep sequence models, but in evaluating a parsimonious surrogate-modeling framework tailored to data-scarce settings and reproducible decision-support workflows. Within this specific context, the study compares bagging-based and AdaBoost-hybrid learners across multiple lag scenarios and locations, and examines how temporal persistence, irrigation influence, and hydro-climatic heterogeneity affect surrogate-model skill. The resulting contribution is methodological and comparative: it clarifies where simple interpretable ensemble surrogates are adequate, where they begin to fail, and why these limits matter when reproducing GEOS-derived SSW dynamics.

This study is best understood as a methodological case study that evaluates machine learning models for surrogate prediction of NASA POWER (GEOS-derived) soil surface wetness (SSW) across a limited number of selected sites in Punjab, north-western India. Rather than emphasizing broad novelty, the contribution lies in comparing AdaBoost-based hybrid models and bagging ensembles as computationally efficient surrogate approaches to reproduce modeled SSW dynamics across multiple lag-based input scenarios and feature selection schemes. The analysis also examines site-specific differences in model performance, providing limited but useful methodological evidence on how surrogate-model behavior may vary under contrasting hydro-climatic conditions. Accordingly, the findings are intended as comparative guidance for surrogate wetness modeling within the study context, rather than as broadly generalizable conclusions for regional soil wetness prediction. This study evaluates ten machine-learning models, including five base models-RF, XGBoost, LightGBM, LMT, and CART and five AdaBoost-based hybrid variants: AdaBoost-RF, AdaBoost-XGBoost, AdaBoost-LightGBM, AdaBoost-LMT, and AdaBoost-CART to identify effective approaches for emulating and predicting monthly NASA POWER (GEOS-derived) soil surface wetness (SSW) at the district scale. The findings are intended to provide a computationally efficient surrogate modeling framework for generating consistent SSW time series that can be readily used in rapid screening, scenario analysis, and decision-support workflows. Such POWER-consistent wetness proxies can assist farmers, land managers, and policymakers in data-driven planning for resource optimization and sustainable land use in data-scarce, environmentally sensitive agro-ecosystems.

## Study area and data source/collection

### Study area

**Fig. 1:**
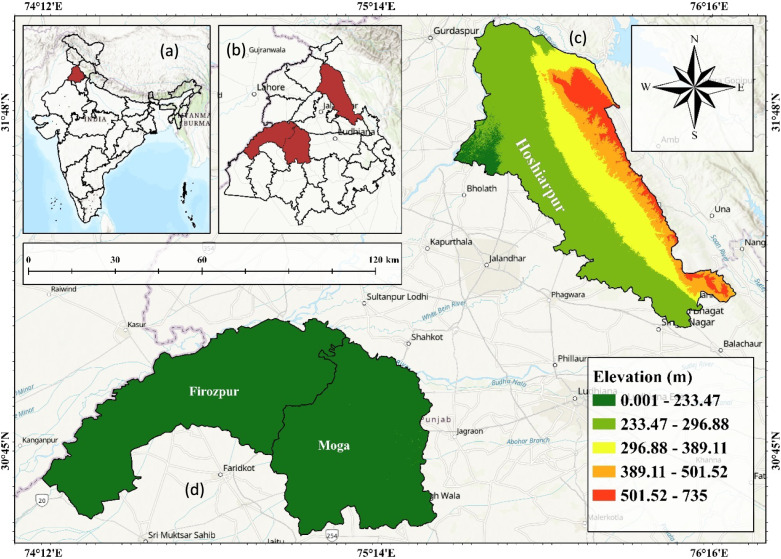
Map of proposed study area (**a**) India Map (**b**) study sites (**c**) Elevation map of Hoshiarpur (**d**) Elevation map of Firozpur and Moga.

The study was conducted across three selected sites in Punjab, north-western India, namely Moga, Hoshiarpur, and Firozpur, representing plain agro-climatic settings, with Hoshiarpur lying closer to the Shivalik foothill transition (Fig. 1). The latitude and longitude of the study area are 30.816° N, 75.171° E for Moga, 31.514° N, 75.911° E for Hoshiarpur, and 30.923° N, 74.610° E for Firozpur. The elevation of the study area ranges from 0.001 m to 735 m.

In Moga City, wheat and rice are the main crops, and rice requires more water than wheat in all climatic zones. This means that crop production depends on the available SSW in the region. The climate of Moga City can be classified as hot semi-arid. The average annual rainfall (AAR) in Moga City is 398 mm, and the mean temperature is around 12 °C, ranging from about 13 °C in winter to 24 °C in summer. Relative humidity is typically high in the morning, exceeding 70%, except in summer, when it falls below 50%. The humidity is comparatively lower in the afternoons. The elevations of Moga city range from about 305 m above sea level in the North-East to about 213 m above sea level in the South-West, with a gentle slope of about 1 m in 1.6 km.

The AAR in the Hoshiarpur location is 938 mm. Most rainfall occurs during the monsoon season, accounting for approximately 77% of the annual total. The climate in Hoshiarpur is humid subtropical, with a dry-winter regime, and it is milder than that of other districts in the Punjab state, with many hills and forests. The main occupation in the area is farming. Most of the physiographic units in the area are categorized as Siwalik hill top and slopes (eroded), Choe belt upper piedmont plain, lower piedmont plain, recent flood plain, active flood plain and old flood plain. The temperature at Hoshiarpur ranges from 5.6 °C to 40.2 °C (NBSSLUP, Report No. 485). The geological formations at Hoshiarpur belong to the Indo-Gangetic Plain, which formed during the Pleistocene. Part of the area is covered by Siwaliks, which consist mainly of Upper Tertiary River deposits and alluvial materials transported from surrounding mountains. The geology of the outer Siwalik range comprises beds of sand alternating with loam, clay, and loose gravel beds (NBSSLUP, report no.485).

The total geographical area of Firozpur district is 5,850 km^2^, the district is a part of Sutlej River Basin, and AAR is 389 mm. The climate is semi-arid, with distinct seasons. The major physiographic units of the district include upland plains, sand dune tracts, younger floodplains, and active floodplains. Land use and land cover include forested areas and agricultural fields dominated by rice and wheat. The soils are mainly of two types: sierozem in the north and desert soils in the south. The major sources of water-bearing formations are sand and gravel. During the pre-monsoon season, the water level varies from 0.73 to 11.35 m below groundwater level (bgl). During the post-monsoon season, water level varies from 0.49 to 9.60 m bgl, with a long-term declining trend of about 0.43 m per year.

The three study locations-Moga, Hoshiarpur, and Firozpur-were selected to represent contrasting agro-climatic and land-management conditions within Punjab, north-western India. Hoshiarpur, located closer to the Shivalik foothills, generally experiences relatively higher rainfall and greater climatic influence from orographic processes. Moga represents a central plain region with moderate rainfall and mixed irrigation dependence, while Firozpur, situated in the south-western plains, is characterized by lower and more variable precipitation, higher evaporative demand, and intensive canal- and groundwater-based irrigation.

This deliberate selection enables evaluation of model performance across a gradient of hydro-climatic persistence, irrigation dominance, and land-surface heterogeneity, thereby facilitating a comparative assessment of surrogate modeling robustness under differing conditions. While the three stations do not capture the full spatial heterogeneity of the region, they are representative of key agro-climatic zones within Punjab and are sufficient for drawing methodological and relative performance conclusions regarding the behaviour of hybrid ensemble models. Accordingly, regional-scale implications are interpreted in terms of comparative trends and model applicability rather than direct spatial extrapolation.

### Data source/collection

The soil surface wetness (SSW) data used in this study were obtained from the NASA POWER platform, which provides GEOS-based land-surface model outputs at a spatial resolution of 0.5° × 0.625°. Direct validation against long-term in-situ soil moisture observations was not performed due to the lack of consistent, site-level measurements for the study locations. Accordingly, the NASA POWER SSW dataset was treated as a model-derived reference, and the analysis focuses on emulating its temporal dynamics rather than validating true ground-measured wetness. The relatively coarse spatial resolution of the NASA POWER SSW product may not fully capture site-scale heterogeneity arising from variations in soil texture, land use, irrigation practices, and micro-topography. This scale mismatch can introduce smoothing effects and may partially explain reduced model skill in locations dominated by strong management-driven or short-term variability. No spatial downscaling or interpolation techniques were applied in the present study in order to maintain internal consistency with the source dataset and the surrogate-modeling objective. The SSW dataset was obtained from the NASA POWER website (https://power.larc.nasa.gov/data-access-viewer/). To characterize the temporal behaviour of soil surface wetness (SSW), several descriptive statistical measures were derived and analyzed. SSWmin (soil surface wetness minimum) represents the minimum wetness value and reflects the driest surface conditions, which are typically associated with prolonged dry periods, high evaporative demand, or limited precipitation and irrigation inputs. SSWmax (soil surface wetness maximum) denotes the maximum wetness value and corresponds to peak surface saturation conditions following rainfall or irrigation events.

SSWmean (soil surface wetness mean) provides the long-term average wetness level and serves as an indicator of the overall moisture regime at a location, integrating the combined effects of climate, soil properties, and land management. SSWsd (soil surface wetness standard deviation) quantifies the variability of SSW around its mean and reflects the degree of temporal fluctuation in surface wetness, with higher values indicating more dynamic wetting–drying cycles. Higher-order statistical moments were also considered to describe the distributional characteristics of SSW. SSWskw (soil surface wetness skewness) measures the asymmetry of the SSW distribution and provides insight into whether wetness dynamics are dominated by frequent dry conditions with occasional wet extremes or vice versa. SSWkrt (soil surface wetness kurtosis) describes the peakedness and tail behavior of the distribution, indicating the relative prevalence of extreme wetness events compared to more moderate conditions (Table 1). The SSWmin it varies from 0.09 to 0.35, SSWmax from 0.09 to 0.66, SSWmean from 0.29 to 0.66, SSWsd from 0.10 to 0.11, SSWkrt from − 0.35 to 0.09 and SSWskw − 0.02 to 0.34 of Moga location.

The soil surface wetness (SSW) dataset used for model development was obtained from the NASA Prediction of Worldwide Energy Resources (POWER) platform. It should be noted that NASA POWER does not provide direct observational soil moisture measurements. Instead, the SSW data are generated using the Goddard Earth Observing System (GEOS) global circulation model, which integrates satellite observations, land surface parameterizations, and atmospheric reanalysis products. Therefore, the SSW values represent model-derived estimates rather than in situ ground truth data. In this context, the machine learning models developed in the present study are trained and validated against GEOS-simulated SSW outputs. The objective is to assess the capability of ensemble and hybrid machine learning techniques to act as surrogate models that emulate the underlying physical model behavior with improved computational efficiency and predictive consistency.

**Fig. 2:**
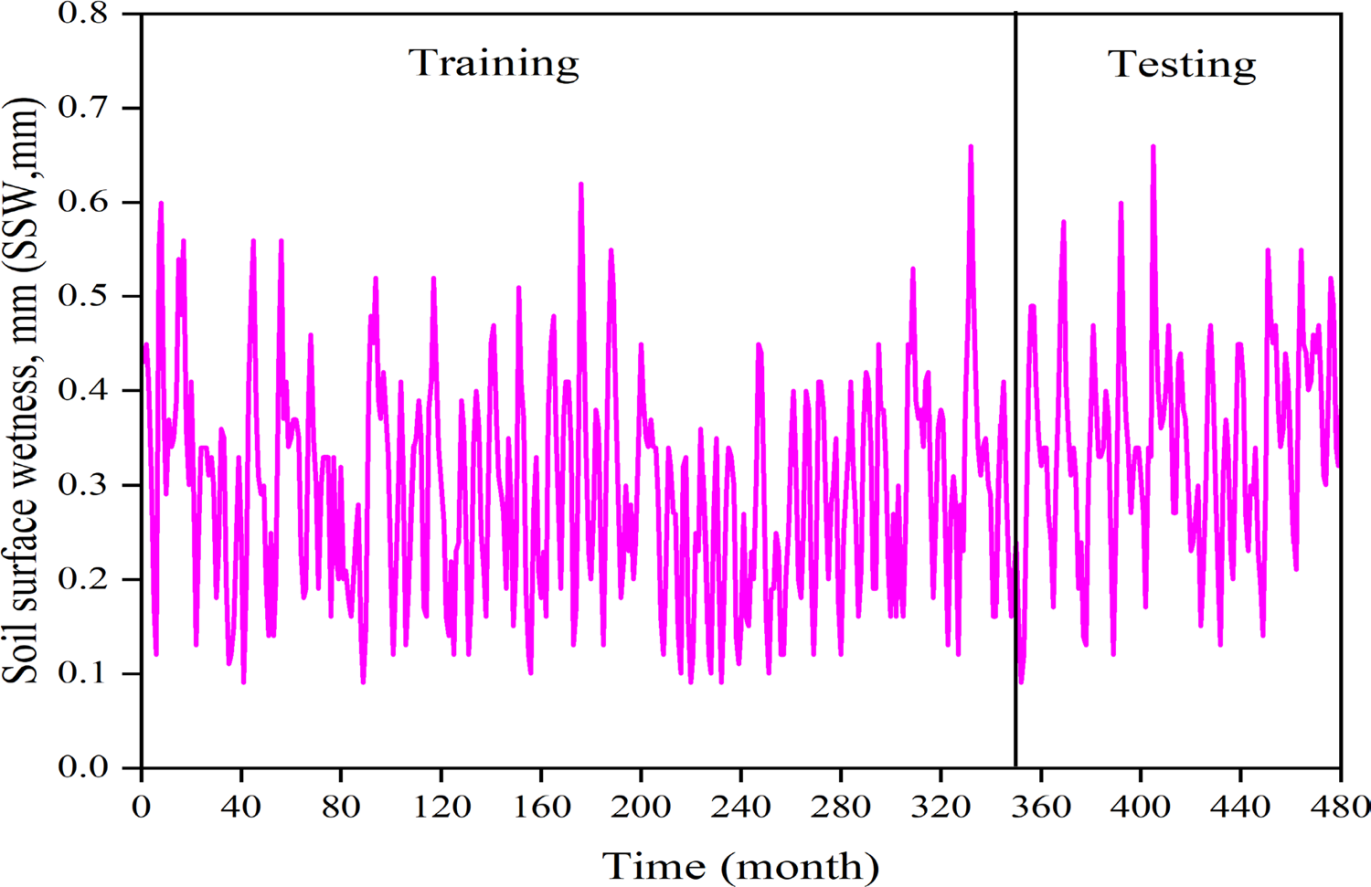
Length of data set utilize for proposed study area.

Similarly, in Hoshiarpur, SSWmin varies from 0.09 to 0.12, SSWmax from 0.74 to 0.79, SSWmean from 0.33 to 0.43, SSWsd from 0.13 to 0.14, SSWkrt from − 0.29 to 0.38, and SSWskw from 0.18 to 0.51. In Firozpur, SSWmin ranges from 0.09 to 0.30, SSWmax from 0.09 to 0.73, and SSWmean from 0.28 to 0.73. The dataset patterns for the proposed study area are shown in Fig. 2.

## Proposed methodology

### Random forest (RF)

Random forest (RF) is an ensemble machine learning algorithm used for classification and regression tasks. It constructs multiple decision trees during training and combines their outputs to improve predictive accuracy and reduce overfitting. In classification problems, the final prediction is typically made through majority voting among the individual trees, while in regression tasks, the average prediction is used. Random forest introduces randomness by selecting bootstrap samples from the original dataset and considering a random subset of features at each split, which enhances model diversity and robustness^31^. It can handle both categorical and numerical data, is relatively tolerant to noise and missing values, and performs well with large and complex datasets. Owing to its strong predictive performance and ability to estimate variable importance, random forest is widely applied in data mining and classification studies^32,33^.

### XGBoost

Extreme gradient boosting (XGBoost) is a supervised machine learning algorithm widely used for classification and regression problems. It is based on the gradient boosting framework, in which multiple decision trees are built sequentially, and each subsequent tree attempts to correct the errors of the previous ones. Unlike a single decision tree model, XGBoost combines boosting with regularization, which improves predictive accuracy while reducing the risk of overfitting. The algorithm is designed for speed and performance, with features such as parallel processing, sparsity-aware learning, and optimized tree construction. It can effectively handle large, complex, and high-dimensional datasets, making it one of the most widely applied algorithms in predictive modeling and data mining studies. Due to its efficiency, flexibility, and strong predictive capability, XGBoost has become a preferred machine learning technique in many research domains^34^.

### Light GBM

Light gradient boosting machine (LightGBM) is a supervised machine learning algorithm developed for efficient and high-performance classification and regression. It is a gradient boosting framework that builds decision-tree ensembles sequentially, where each new tree is trained to reduce the errors of the preceding trees. Unlike many traditional boosting methods that grow trees level-wise, LightGBM uses a leaf-wise growth strategy, which can achieve lower loss and faster convergence under the same number of leaves. It also employs histogram-based decision tree learning to improve computational speed and memory efficiency, making it suitable for large-scale and high-dimensional datasets. In addition, LightGBM introduces techniques such as gradient-based one-side sampling (GOSS) and exclusive feature bundling (EFB), which further enhance training efficiency without substantial loss of accuracy. Due to its speed, scalability, and strong predictive performance, LightGBM is widely used in machine learning and data mining applications^35^.

### Logistic model trees (LMT)

Logistic model trees (LMT) is a type of decision tree where each leaf is associated with a logistic regression model. This combines the interpretability of decision trees with the flexibility of logistic regression^36^. This hybrid approach combines the interpretability of decision trees with the flexibility and power of logistic regression. The algorithm works by recursively splitting the dataset into subsets based on certain features, similar to traditional decision trees. However, instead of predicting a class directly at the leaves, each leaf corresponds to a logistic regression model. This model is then used to estimate the probability of belonging to a particular class^37^. LMTs are particularly useful for datasets that exhibit complex relationships, as they can capture nonlinear patterns and interactions among features. The logistic regression models at the leaves enable more nuanced predictions, making LMTs suitable for both classification and regression tasks^36^. This approach is advantageous in situations where a simple decision tree may not capture the intricacies of the underlying data distribution.

### Classification and regression tree (CART)

CART stands for classification and regression trees. It’s a decision tree algorithm that can be used for both classification and regression tasks. CART constructs binary trees and is known for its simplicity and effectiveness^16,38^. By constructing binary trees through recursive splitting of the dataset based on feature values, CART creates a hierarchical decision-making structure^39^. It determines the optimal features and thresholds for splitting nodes using measures such as Gini impurity or information gain. In regression-mode CART, node splitting is performed by minimizing the sum of squared errors (SSE) for the resulting child nodes^40^.

1 $$\mathrm{argmin} [{P}_{i}Var\left({Y}_{i}\right)+{P}_{r}Var({Y}_{r})], {x}_{j}\le {x}_{j}^{R},J=1\dots \dots \dots \dots \dots M$$

where Var (Yi) and Var(Yr) are response variables for corresponding child nodes of a parent node, xj is variable j; ${x}_{j}^{R}$ is the best splitting value of variable xj; M is number of variables xj.${x}_{j}^{R}$ and j = 1…..; and M is an optimal splitting question which satisfies Eq. (1). The squared residuals minimization algorithm is identical to the Gini splitting rule of Gini index. It is used as the following impurity measure i(t):

2 $$\mathrm{i}\left(\mathrm{t}\right)=1-{\sum}_{k=1}^{k}{p}^{2}\left(k|t\right)$$

where k, I1,….; K is an index of the class, and $p\left(k|t\right)$ is conditional probability of class k, provided in node t. CART accommodates both categorical and numeric attributes, employing pruning techniques to prevent overfitting and enhance generalization to unseen data^41^. Often serving as a foundational model in ensemble methods like random forests, CART’s interpretability and capability to handle various data types make it widely adopted across domains, providing insights into decision-making processes while delivering robust predictive performance^42^. CART accommodates both categorical and numeric attributes, employing pruning techniques to prevent overfitting and enhance generalization to unseen data. Often serving as a foundational model in ensemble methods such as random forests, CART’s interpretability and ability to handle diverse data types make it widely adopted across domains, providing insights into decision-making processes while delivering robust predictive performance^43^. The various parameter tuning used to develop the models are described in Table 1.

**Table 1:** Descriptive statistics of Moga, Hoshiarpur and Firozpur locations.

Statistical parameter	Total data sets	Training data sets (70%)	Testing data sets (30%)	Total data sets	Training data sets (70%)	Testing data sets (30%)
Descriptive statistics of Moga location						
SSWM (t)				SSWM (t-1)		
SSWmin	**0.09**	**0.09**	**0.12**	**0.09**	**0.09**	**0.12**
SSWmax	**0.66**	**0.66**	**0.66**	**0.66**	**0.66**	**0.66**
SSWmean	0.31	0.29	0.35	0.31	0.29	0.35
SSWsd	0.11	0.11	0.11	0.11	0.11	0.11
SSWkrt	**− **0.35	**− **0.35	**− **0.01	**− **0.35	**− **0.35	0.00
SSWskw	0.19	0.29	0.08	0.20	0.29	0.10
SSWM (t-2)				SSWM (t-5)		
SSWmin	0.09	0.09	0.12	0.09	0.09	0.12
SSWmax	0.31	0.29	0.35	0.31	0.29	0.35
SSWmean	0.66	0.66	0.66	0.66	0.66	0.66
SSWsd	0.11	0.11	0.11	0.11	0.11	0.10
SSWkrt	**− **0.34	**− **0.35	0.03	**− **0.28	**− **0.25	0.08
SSWskw	0.20	0.29	0.11	0.21	0.32	0.10
SSWM (t-6)				SSWM (t-9)		
SSWmin	0.09	0.09	0.12	0.09	0.09	0.12
SSWmax	0.31	0.29	0.35	0.31	0.29	0.35
SSWmean	0.66	0.66	0.66	0.66	0.66	0.66
SSWsd	0.11	0.11	0.10	0.11	0.11	0.11
SSWkrt	**− **0.28	**− **0.24	0.07	**− **0.30	**− **0.24	0.09
SSWskw	0.22	0.33	0.10	0.22	0.34	**− **0.02
SSWM (t-11)				SSWM (t-12)		
SSWmin	0.09	0.09	0.12	0.09	0.09	0.12
SSWmax	0.31	0.29	0.35	0.31	0.29	0.35
SSWmean	0.66	0.66	0.66	0.66	0.66	0.66
SSWsd	0.11	0.11	0.11	0.11	0.11	0.11
SSWkrt	**− **0.32	**− **0.27	0.00	**− **0.32	**− **0.29	**− **0.04
SSWskw	0.21	0.32	**− **0.01	0.21	0.31	0.02
Descriptive statistics of Hoshiarpur location						
SSWM (t)				SSWM (t-1)		
SSWmin	0.09	0.09	0.12	0.09	0.09	0.12
SSWmax	0.79	0.78	0.79	0.79	0.78	0.79
SSWmean	0.36	0.33	0.43	0.36	0.33	0.43
SSWsd	0.14	0.13	0.14	0.14	0.13	0.14
SSWkrt	0.10	0.27	**− **0.24	0.11	0.27	**− **0.24
SSWskw	0.48	0.47	0.36	0.48	0.48	0.37
SSWM (t-2)				SSWM (t-5)		
SSWmin	0.09	0.09	0.12	0.09	0.09	0.12
SSWmax	0.79	0.78	0.79	0.78	0.78	0.74
SSWmean	0.36	0.33	0.43	0.36	0.33	0.43
SSWsd	0.14	0.13	0.14	0.14	0.13	0.13
SSWkrt	0.13	0.27	**− **0.22	0.07	0.38	**− **0.29
SSWskw	0.48	0.47	0.39	0.44	0.50	0.30
SSWM (t-6)				SSWM (t-9)		
SSWmin	0.09	0.09	0.12	0.09	0.09	0.09
SSWmax	0.78	0.78	0.74	0.78	0.78	0.74
SSWmean	0.36	0.33	0.43	0.36	0.33	0.42
SSWsd	0.14	0.13	0.13	0.14	0.13	0.14
SSWkrt	0.07	0.36	**− **0.24	0.06	0.38	**− **0.13
SSWskw	0.44	0.50	0.31	0.43	0.51	0.18
SSWM (t-11)				SSWM (t-12)		
SSWmin	0.09	0.09	0.09	0.09	0.09	0.09
SSWmax	0.78	0.78	0.74	0.78	0.78	0.74
SSWmean	0.36	0.33	0.42	0.36	0.33	0.42
SSWsd	0.14	0.13	0.14	0.14	0.13	0.14
SSWkrt	0.07	0.35	**− **0.15	0.09	0.34	**− **0.15
SSWskw	0.43	0.49	0.21	0.43	0.48	0.23
Descriptive statistics of Firozpur location						
SSWM (t)				SSWM (t-1)		
SSWmin	0.09	0.56	0.73	0.09	0.56	0.73
SSWmax	0.30	0.09	0.12	0.30	0.09	0.12
SSWmean	0.73	0.28	0.33	0.73	0.28	0.33
SSWsd	0.11	0.11	0.12	0.11	0.11	0.12
SSWkrt	**− **0.32	**− **0.74	0.21	**− **0.32	**− **0.74	0.20
SSWskw	0.40	0.34	0.50	0.39	0.33	0.50
SSWM (t-2)				SSWM (t-5)		
SSWmin	0.09	0.56	0.73	0.09	0.56	0.73
SSWmax	0.30	0.09	0.12	0.30	0.09	0.12
SSWmean	0.73	0.28	0.33	0.62	0.28	0.33
SSWsd	0.11	0.11	0.12	0.11	0.11	0.11
SSWkrt	**− **0.33	**− **0.75	0.20	**− **0.61	**− **0.71	**− **0.49
SSWskw	0.39	0.32	0.51	0.32	0.33	0.30
SSWM (t-6)				SSWM (t-9)		
SSWmin	0.09	0.56	0.73	0.09	0.56	0.73
SSWmax	0.30	0.09	0.12	0.30	0.09	0.12
SSWmean	0.62	0.28	0.33	0.62	0.28	0.32
SSWsd	0.11	0.11	0.11	0.11	0.11	0.11
SSWkrt	**− **0.67	**− **0.66	**− **0.63	− 0.67	− 0.66	− 0.58
SSWskw	0.30	0.36	0.22	0.30	0.37	0.17
SSWM (t-11)				SSWM (t-12)		
SSWmin	0.09	0.56	0.73	0.09	0.56	0.73
SSWmax	0.30	0.09	0.12	0.30	0.09	0.12
SSWmean	0.62	0.28	0.32	0.62	0.28	0.32
SSWsd	0.11	0.11	0.11	0.11	0.11	0.11
SSWkrt	− 0.68	− 0.70	− 0.58	− 0.68	− 0.71	− 0.56
SSWskw	0.30	0.35	0.20	0.30	0.34	0.23

### Adaptive boosting (AdaBoost) model

**Fig. 3:**
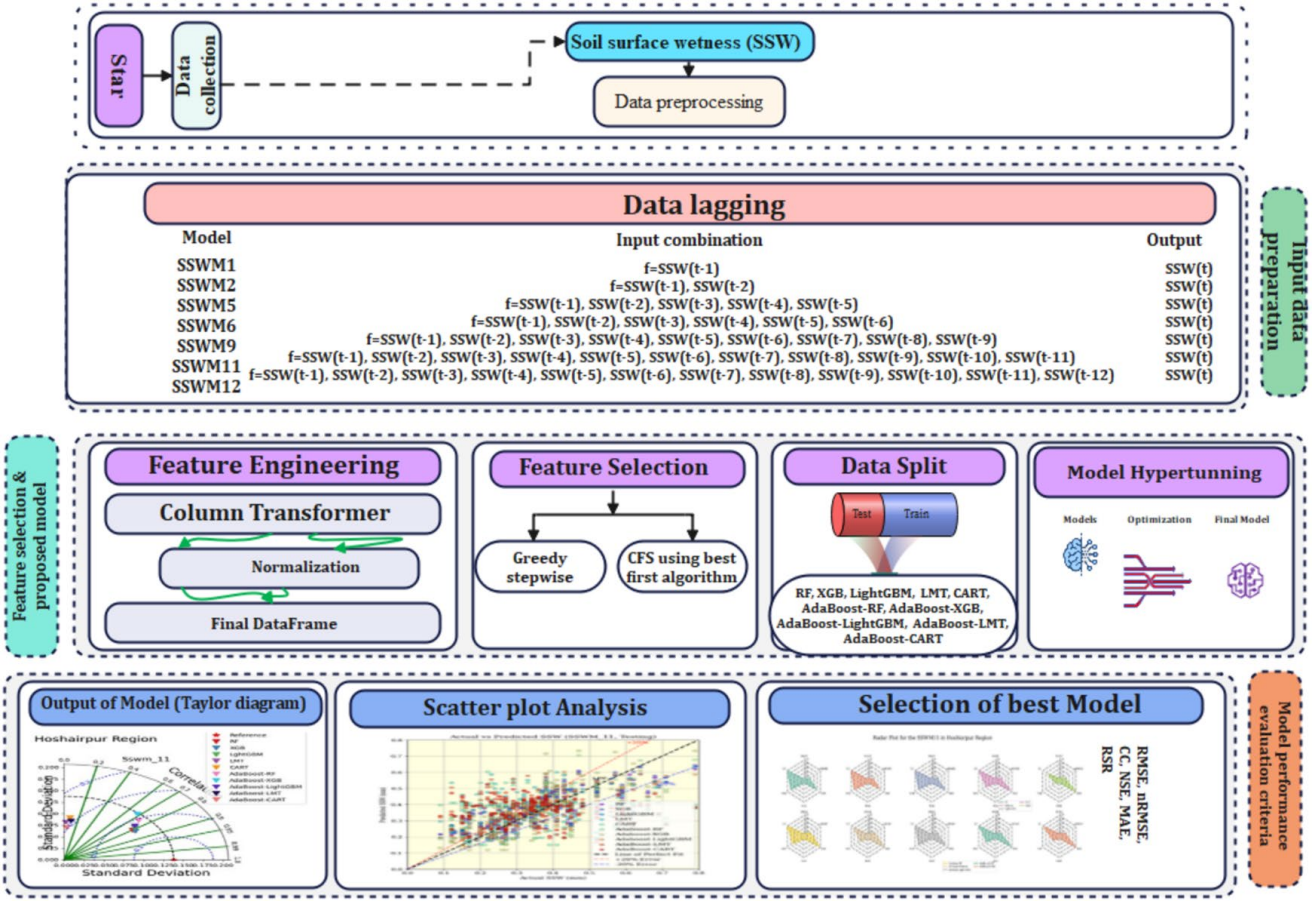
Flow chart of proposed methodology.

AdaBoost model is developed by Freund and Schapire^44^ to improve the accuracy of the classification-based ML models. AdaBoost is an ensemble boosting algorithm originally developed to improve weak learners through iterative reweighting of difficult cases. In the present study, AdaBoost was used in a regression-oriented framework to enhance the predictive performance of the selected base learners for continuous soil surface wetness (SSW) estimation. Previous studies have examined AdaBoost in several hydrological applications, however, its performance for SSW prediction remains underexplored^45^. The details of the proposed methodology are mentioned in Fig. 3.

### Proposed methodology for model development

A data-lagging approach was used to develop monthly soil surface wetness (SSW) prediction models for the study area. Monthly NASA POWER (GEOS-derived) SSW time-series data were used for model development. The input–output combinations considered in the developed models are presented in Table 2. The full dataset was divided chronologically into training and testing subsets, with 70% of the observations used for model training and the remaining 30% used for model testing. Lagged-input cases were constructed using antecedent SSW values from 1 to 12 months prior to the prediction time step. Among these, the selected model structures included Soil Surface Wetness Model 1 (SSWM1), SSWM2, SSWM5, SSWM6, SSWM9, SSWM11, and SSWM12, as described in Table 2.

**Table 2:** Input and output combination of parameters are used for model development.

Model	Input combinations	Output
SSWM 1	*f=SSW(t-1)*	SSW1
SSWM 2	$f=SSW\left(t-1\right), SSW(t-2)$	SSW 2
SSWM 5	$f=SSW\left(t-1\right), SSW\left(t-2\right),SSW\left(t-3\right),SSW\left(t-4\right),SSW(t-5)$	SSW(t)
SSWM 6	$f=SSW\left(t-1\right),SSW\left(t-2\right),SSW\left(t-3\right), SSW\left(t-4\right), SSW\left(t-5\right), SSW(t-6)$	SSW(t)
SSWM 9	$f=SSW\left(t-1\right), SSW\left(t-2\right), SSW\left(t-3\right), SSW\left(t-4\right), SSW\left(t-5\right), SSW\left(t-6\right), SSW\left(t-7\right),SSW\left(t-8\right),SSW(t-9)$	SSW(t)
SSWM 11	$f=SSW\left(t-1\right), SSW\left(t-2\right),SSW\left(t-3\right),SSW\left(t-4\right), SSW\left(t-5\right),SSW\left(t-6\right), SSW\left(t-7\right), SSW\left(t-8\right), SSW\left(t-9\right), SSW\left(t-10\right),SSW(t-11)$	SSW(t)
SSWM 12	$f=SSW\left(t-1\right), SSW\left(t-2\right), SSW\left(t-3\right), SSW\left(t-4\right), SSW\left(t-5\right),SSW\left(t-6\right),SSW\left(t-7\right),SSW\left(t-8\right),SSW\left(t-9\right), SSW\left(t-10\right), SSW\left(t-11\right), SSW(t-12)$	SSW(t)

*SSWM* Soil surface wetness model.

In this study, soil surface wetness (SSW) was modeled as a continuous response variable, and all machine learning algorithms were implemented in regression mode. No discretization, binning, or categorical transformation of SSW was performed at any stage of data pre-processing, feature selection, model training, or evaluation. Although some of the applied algorithms, such as RF, XGBoost, LightGBM, LMT, and CART, are widely used in predictive modeling, in this study they were configured as regression-based learners to enable the direct prediction of continuous SSW values. Model performance was therefore assessed using standard regression metrics (e.g., RMSE, nRMSE, NSE, RSR, and CC), consistent with continuous-variable prediction. The selected algorithms were chosen to match the structure and objective of the present study rather than to represent an exhaustive comparison of all available machine-learning methods. The target variable was monthly NASA POWER (GEOS-derived) soil surface wetness, and the predictors consisted only of lagged SSW values; therefore, the modeling task was a low-dimensional, autoregressive surrogate-learning problem with a relatively limited sample size and a strong need for interpretability. Under such conditions, tree-based and tree-hybrid methods are appropriate because they can represent nonlinear responses, threshold effects, and regime-dependent behavior without requiring strong distributional assumptions. CART was employed as a simple variance-minimizing regression-tree baseline, whereas LMT was included to examine the potential advantage of combining tree-structured partitioning with model-based learning under the lag-based predictor setting. RF, XGBoost, and LightGBM were incorporated to evaluate the ability of ensemble and gradient-boosting approaches to capture complex nonlinear patterns and variable interactions in SSW. In addition, their AdaBoost-based hybrid variants were evaluated to assess whether further boosting improves predictive accuracy beyond that of the individual base learners.

In the present monthly GEOS-derived SSW series, this is useful for identifying data-supported transitions among persistent dry states, transitional recharge phases, and wetter conditions, rather than imposing arbitrary threshold splits. LMT was selected because monthly SSW does not evolve only through abrupt threshold changes. Part of the signal reflects gradual atmosphere-driven wetting–drying behaviour associated with seasonal rainfall progression, evapotranspiration, and storage depletion, whereas another part reflects sharper departures caused by episodic rainfall or irrigation inputs. The tree component partitions the data into broad wetness regimes, while the local piecewise model within each terminal node captures smoother within-regime variation. This makes LMT suitable for representing gradual seasonal evolution while still accommodating abrupt wetness shifts through regime partitioning. AdaBoost versions of these learners were then introduced to test whether sequential emphasis on poorly predicted instances improves surrogate prediction of GEOS-derived SSW, particularly during seasonal transitions, wetness extremes, and heterogeneous response conditions. Thus, the model set was intentionally designed to compare interpretable single-tree and hybrid boosted learners under the same lag-based framework and to assess whether added ensemble complexity yields meaningful gains for monthly SSW surrogate modeling. A chronological 70–30% train–test split was employed to evaluate model performance, with the earlier portion of the time series used for training and the later portion reserved for testing. This approach preserves temporal ordering and avoids information leakage from future observations into model calibration, which is essential for time-dependent hydrological variables such as soil surface wetness. Feature selection was conducted using cross-validation within the training period to improve robustness and reduce overfitting. While this validation strategy provides a clear and reproducible baseline assessment, more advanced time-series validation schemes (e.g., rolling-origin or blocked cross-validation) are recommended for future work to further evaluate temporal generalizability under non-stationary conditions. Thus, the lag-only input structure was intended to test the predictive value of antecedent SSW memory, but it also represents a structural limitation where wetness dynamics are governed by factors not encoded in prior SSW values alone.

### Best input selection criteria of the proposed model

Feature selection and construction, also known as feature extraction, refers to the process of extracting new features by transforming the original sample data set representation, thereby projecting the problem into a more discriminative (informative) space that makes the classification task more efficient^46^. The objective of feature selection is to find the optimal subset consisting of m features chosen from the total n features. The advantages of filter-based feature selection techniques are their speed and interpretability. In the present study, Best-First and greedy stepwise search techniques were employed. Greedy stepwise offers the advantage of simplicity and fast solution search, however, it may yield suboptimal solutions and is prone to false starts due to its dependence on local decisions^46,47^. The most common feature selection criteria use forward or backward strategies, which always provide a best subset optimal solution.

For feature selection, two algorithms are applied, specifically the best first and greedy stepwise methods. First, we applied a greedy stepwise search algorithm that relies on an attribute evaluator and a search method (Table 3). Attribute evaluator has two sub-sections: the wrapper subset evaluator and the classifier. M5Rules was used in the feature-selection stage because it is suitable for continuous-response problems and provides a practical basis for assessing the relative relevance of lagged predictors within a regression-oriented framework. In the M5Rules algorithm, several parameters are used, such as batch size (set to 100) and a minimum number of instances (set to 4). Similarly, for the wrapper subset evaluator, different parameters are evaluated: feature measures; folds are set to 5; the seed is 1; and the threshold value is 0.01. In the search method, the greedy stepwise algorithm is utilized for feature selection. In this algorithm, several parameters are applied, such as forward selection, ranking method, number of execution slots, number of attributes to retain (set to 1), and the threshold was set to a very low value (i.e., effectively no threshold), ensuring that no attributes were excluded a priori during the selection process. Therefore, the attribute selection mode process used data sets, 20 folds, and seed 1 for cross-validation and for feature selection. The greedy stepwise method selected SSW(t–1), SSW(t–2), SSW(t–5), SSW(t–11), and SSW(t–12) as the most relevant predictors, achieving an overall correlation coefficient (CC) of 0.75. Second, we have applied the best first algorithm based on correlation-based feature selection (CFS) of the proposed study area.

**Table 3:** Selected feature based on greedy stepwise method.

Number of folds	Percentage (%)	Attribute
16	80	1 SSW(t-12)
20	100	2 SSW(t-11)
0	0	4 SSW(t-9)
6	30	7 SSW(t-6)
20	100	8 SSW(t-5)
20	100	11 SSW(t-2)
20	100	12 SSW(t-1)

Best first search is an artificial intelligence strategy that allows backtracking along the search path. Like greedy hill climbing, best first moves through the search space by making local changes to the current feature subset^48^. In this algorithm, the parameter attribute evaluator is used. The number of threads to use is 1 and the pool size is set to 1. Additionally, in the search method, the best-first algorithm is used; for this technique, parameters are evolved, such as forward direction, lookup cache size equal to 1, and search termination is 5. The CC of the best first algorithm found is 0.67; similarly, cross-validation is also applied to check the accuracy of algorithms. In this cross-validation, values of 20 folds and seed = 1 are used for evaluation of feature selection for the proposed study area, as shown in Table 4.

**Table 4:** Selected feature CFS using best first algorithm.

Number of folds	Percentage (%)	Attribute
20	100	1 SSW(t-12)
0	0	2 SSW(t-11)
20	100	4 SSW(t-9)
2	10	7 SSW(t-6)
18	90	8 SSW(t-5)
0	0	11 SSW(t-2)
20	100	12 SSW(t-1)

While greedy stepwise and best first (CFS) feature selection methods were employed in this study due to their interpretability and effectiveness in reducing redundancy among lagged predictors, alternative feature selection strategies may offer additional advantages. Information-theoretic approaches such as minimum redundancy maximum relevance (MRMR) and mutual information–based selection are capable of capturing nonlinear dependencies between predictors and the target variable while explicitly controlling redundancy. Such methods may be particularly useful when extending the current framework to include meteorological forcings or remotely sensed variables, where relationships with soil surface wetness are often nonlinear. Future studies could systematically compare wrapper-based, correlation-based, and information-theoretic feature selection techniques to assess their relative impact on surrogate model performance and robustness.

The lagged predictors selected by the greedy stepwise and best first (CFS) algorithms exhibit clear physical relevance when interpreted in the context of soil–atmosphere interactions. The consistent selection of SSW(t − 1) reflects strong short-term soil moisture persistence, arising from soil water storage, delayed drainage, and gradual evapotranspiration processes that carry wetness information from one month to the next. This short-memory effect is a well-documented characteristic of near-surface soil moisture dynamics. The frequent selection of SSW(t − 12) highlights the importance of seasonal and annual memory, particularly in monsoon-influenced and irrigation-dominated agro-climatic regions. Annual recurrence of rainfall patterns, cropping calendars, and irrigation schedules means that soil wetness conditions in a given month are often related to conditions during the same period in the previous year. The inclusion of this lag enables the models to account for repeating seasonal controls on soil surface wetness. Intermediate lags such as SSW(t − 5), SSW(t − 6), and SSW(t − 9) capture intra-seasonal soil–atmosphere coupling, reflecting cumulative effects of rainfall, evapotranspiration demand, and irrigation within a cropping season. These lags are physically associated with gradual soil moisture recharge and depletion processes rather than instantaneous responses to single precipitation events.

Differences between the greedy stepwise and best first (CFS) selections further reflect physically meaningful redundancy patterns. While greedy stepwise may retain multiple correlated lags representing similar persistence mechanisms, the CFS-based best first approach preferentially selects a smaller subset of predictors that balance physical relevance with reduced redundancy. Together, these results indicate that the selected predictors are not merely statistically optimal but correspond to distinct soil moisture memory processes operating at short-term, intra-seasonal, and annual timescales. The use of 1–12 month lagged predictors was designed to capture multiple temporal memory components in monthly soil surface wetness (SSW), including short-term persistence and seasonal to annual recurrence. Monthly soil moisture and wetness time series are well known to exhibit strong auto-correlation at short lags due to soil water storage and delayed evapotranspiration, as well as pronounced peaks at seasonal or annual scales associated with recurring climatic forcing and land-management cycles. The present study intentionally employed only lagged SSW values as predictors because the goal was to develop a parsimonious surrogate (emulator) model of NASA POWER (GEOS-derived) SSW, rather than a process-explicit hydro-meteorological model incorporating multiple exogenous drivers. This design allows the models to test how effectively antecedent SSW alone can reproduce the temporal persistence and seasonal structure of the GEOS-derived series, although it also limits physical interpretability and may reduce predictive skill where meteorological or site-specific controls are important.

## Model performance analysis of the proposed model

In the present study, several statistical indices were used for model performance, namely root mean square error (RMSE), normalized root mean square error (nRMSE), RMSE-observations standard deviation ratio (RSR), mean absolute error (MAE), nash–sutcliffe efficiency (NSE), and correlation coefficient (CC). Each of the statistical model performance analyses are discussed one by one, and their mathematical expressions are given below.

3 $$\mathrm{C}\mathrm{C}=\frac{\sum_{i=1}^{N}\left({SSW}_{i}-\overline{SSW }\right)\left({SSWP}_{i-}\overline{SSWP }\right)}{\sqrt{\sum_{i=1}^{N}{\left({SSW}_{i}-\overline{SSW }\right)}^{2}{\sum}_{i=1}^{N}{\left(SSW{P}_{i}-\overline{SSWP }\right)}^{2}}}$$

where, ${SSW}_{i}$ is the observed value of SSW, $\overline{SSW }$ is the average value of the observed data, ${SSWP}_{i}$ is the predicted value of the model, $\overline{SSWP }$ is the average value of the predicted value of the model^49^. NSE is commonly used to assess model performance, with values indicating poor, moderate, good, or excellent agreement. NSE ranges from –∞ to 1, where a value of 1 indicates a perfect fit.

4 $$NSE=1-\left(\frac{\sum_{i=1}^{N}{\left({SSW}_{i}-\overline{SSW }\right)}^{2}}{{\sum}_{i=1}^{N}{\left(SSW{P}_{i}-\stackrel{-}{\overline{SSWP} }\right)}^{2}}\right)$$

RMSE is a commonly used performance criterion; lower RMSE values indicate better model performance^50–53^).

5 $$RMSE=\sqrt{\frac{1}{N}{\sum}_{i=1}^{N}{\left({SSW}_{i}-SSW{P}_{i}\right)}^{2}}$$

Normalized RMSE (nRMSE) allows comparison between observed and predicted SSW values by normalizing prediction errors. Its formulation is given in Eq. (6).

6 $$nRMSE= \frac{\sqrt{\frac{1}{N}{\sum}_{i=1}^{N}{({SSW}_{i}-SSW{P}_{i})}^{2}}}{\overline{SSW} }$$

Mean absolute error (MAE) measures the average magnitude of prediction errors between observed and predicted SSW.

7 $$MAE=\frac{1}{N}\sum_{i=1}^{N}\left|{SSW}_{i}-SSW{P}_{i}\right|$$

The RMSE–standard deviation ratio (RSR) is another useful performance index for comparing models^54,55^. RSR ranges from 0 (indicating zero residual variance and perfect model fit) to higher positive values that reflect poorer performance^56^. Lower RSR values correspond to lower RMSE and therefore indicate better agreement between observed and predicted values^57,58^.

8 $$RSR= \frac{RMSE}{{STDEV}_{obs}} = \frac{\sqrt{{{\sum}_{i=1}^{N}({SSW}_{i}-SSW{P}_{i})}_{i}^{2}}}{\sqrt{{{\sum}_{i=1}^{N}({SSW}_{i}-\overline{SSW })}_{i}^{2}}}$$

## Results and discussion

### Results of proposed hybrid models

In the present study, ten models have been applied for the prediction of SSW of the proposed study area. Out of eight models, four bagging-based models and four hybrid models were developed for the computation of SSW of the present study area. Accordingly, model performance in this study should be interpreted as the ability to emulate the temporal dynamics of the GEOS-derived SSW series, rather than as direct evidence of true soil wetness prediction accuracy at the ground level. To evaluate SSW estimation performance, the predictive ability of all ten models-RF, XGBoost, LightGBM, LMT, CART, AdaBoost-RF, AdaBoost-XGBoost, AdaBoost-LightGBM, AdaBoost-LMT, and AdaBoost-CART-was compared. The parameters used in each model are listed in Table 5.

**Table 5:** Number of parameters set used in the proposed models.

S. no	Model	Optimized parameter
1	RF	n estimators: 457, criterion: friedman mse, max depth: 10, min samples split: 10, min samples leaf: 4, max features: 0.474
2	XGBoost	n estimators: 519, max depth: 9, learning rate: 0.493, subsample: 0.553, colsample bytree: 0.89, gamma: 0.051
3	LightGBM	Learning rate: 0.19, num leaves: 226, max depth: 6, min child samples: 37, subsample: 0.506, colsample bytree: 0.51, reg alpha: 0.79, reg lambda: 3.81
4	LMT	Batch size = 100, minimum number of instances = 15, number of boosting iterations = − 1, weight trimming beta = 0
5	CART	Batch size = 100, minimal number of observations = 2, number of folds in the internal cross-validation = 5, seed = 1, percentage of the training set size = 1
6	AdaBoost	Batch size = 100, The number of iterations = 10, seed = 1, Weight threshold for weight pruning = 100

Based on the performance metrics, AdaBoost-LMT generally showed strong relative performance among the tested hybrid models; however, its advantage over standalone LMT was not uniform across all site–scenario combinations. In several cases, the differences between LMT and AdaBoost-LMT were small, and standalone LMT performed comparably or slightly better on individual metrics. This indicates that the benefit of boosting was incremental and context dependent rather than universally dominant across all configurations. Among the three study sites, model performance varied across locations and model configurations; however, Hoshiarpur generally exhibited stronger predictive agreement than Firozpur, while Moga showed intermediate performance in most cases. Integrating LMT into the AdaBoost framework generally improved relative predictive performance, but the gain over standalone LMT was not uniform across all site–scenario combinations. Standalone LMT already performed strongly among the bagging-based models, indicating that its local piecewise structure was well suited to the monthly GEOS-derived SSW signal. The added value of AdaBoost-LMT therefore appears to lie mainly in reducing residual bias and improving representation of difficult transition periods or heterogeneous wetness responses, rather than in producing a universally large performance gain over LMT in every case.

**Fig. 4:**
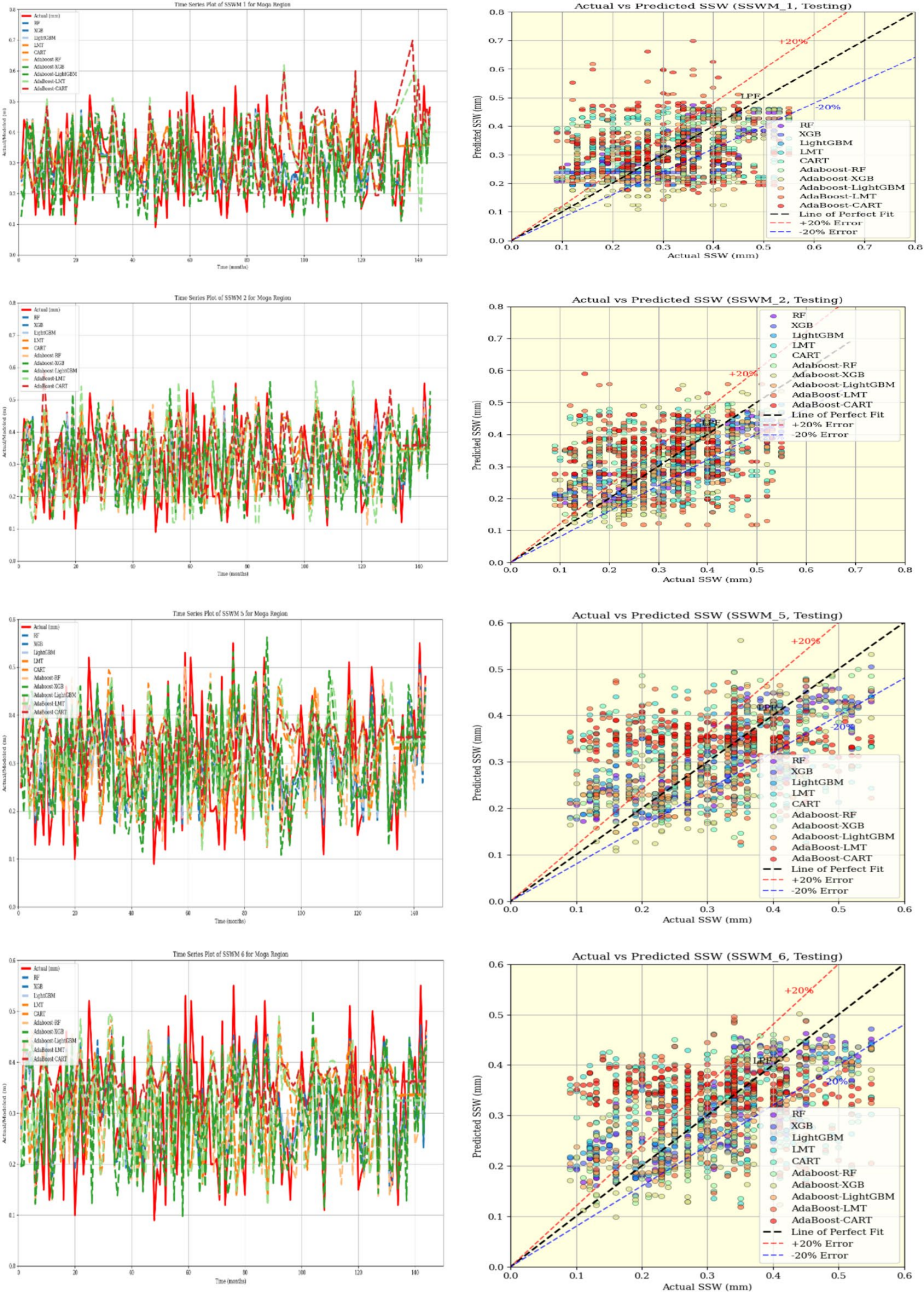

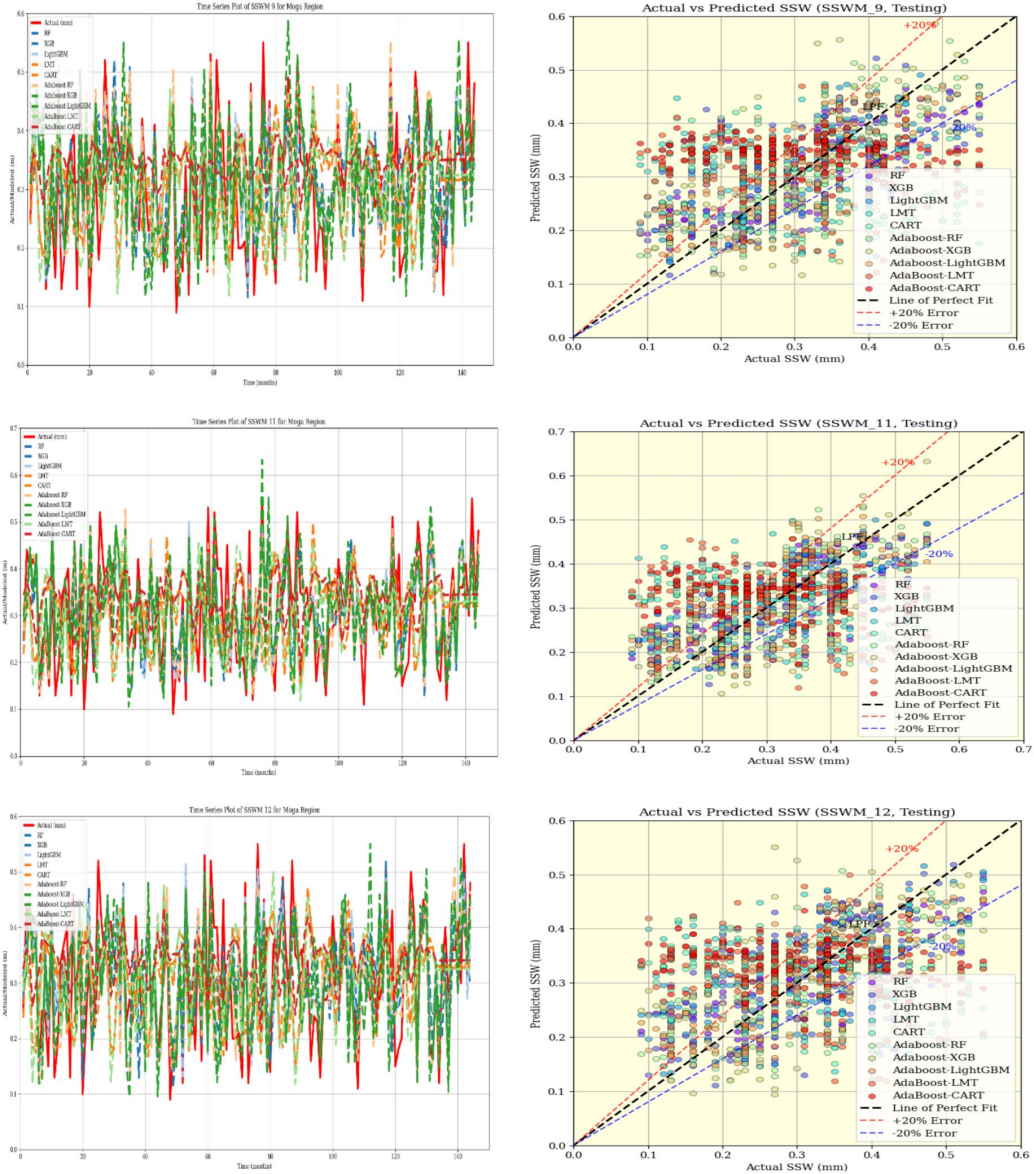
Time series plot of actual and predicted models and scatter plot between actual and predicted SSW of the Moga region during testing of the models.

In Moga, the NSE values for AdaBoost-RF across the SSWM scenarios ranged from 0.61 to 0.76 during the testing period (Fig. 4). SSWM9 produced the highest NSE values among all hybrid model configurations. Therefore, the results of the AdaBoost-LightGBM model of CC vary from 0.61, 0.68, 0.68, 0.72, 0.76, 0.69 to 0.72 for SSWM1, SSWM2, SSWM5, SSWM6, SSWM9, SSWM11, and SSWM12 during the testing of the model. On the basis of the correlation coefficient (CC), it has been observed that the SSWM9 generates better SSW values for the proposed study areas as compared to the other hybrid models.

In terms of error, four statistical techniques were used in the present study area, namely RMSE, nRMSE, MAE, and RSR. The RMSE in the proposed hybrid model varies from 0.09, 0.08, 0.08, 0.08, 0.07, 0.08 to 0.08 for the proposed models. The SSWM2 model produced better RMSE, and SSWM9 gave lower RMSE as compared to the remaining models during the testing of the models. The better RMSE values in SSWM2 and SSWM9 indicate that these input–output combinations are less effective, whereas SSWM5–SSWM6 show only minor variability. This indicates lower uncertainty in SSWM5, SSWM6, SSWM9, SSWM11, and SSWM12. Other error terms as criteria of model performance were evaluated, such as nRMSE. The nRMSE values vary from 0.29, 0.26, 0.26, 0.25, 0.23, 0.26 to 0.25. Based on the nRMSE results, SSWM9 yields satisfactory nRMSE values. Based on nRMSE, we can evaluate the model’s accuracy and suitability in the proposed study area. Therefore, the SSWM1 model generates higher errors, and the SSWM9 gives a slight error in the applied model of the proposed study area. These metrics help normalize and compare errors between observed and predicted SSW. Similarly, the RSR error performance analysis is applied to the proposed model. These criteria combine RMSE and the standard deviation of the observed and predicted values. The RSR can vary from model to model. In the proposed study area, the RSR ranges from 0.80, 0.74, 0.73, 0.70, 0.65, 0.74, to 0.71 for SSWM1 to SSWM12, respectively.

### Results of bagging-based models

The bagging-based models were evaluated using statistical indices to assess their performance in predicting SSW. Four bagging-based models were used to estimate SSW across different input–output combinations. A direct comparison between standalone RF and AdaBoost-LightGBM indicates that the superiority of the boosted version should be interpreted cautiously. RF was already the strongest bagging-based learner across the study sites, which suggests that the tree-plus-local-model structure itself captured an important share of the temporal persistence and regime-dependent variation in GEOS-derived SSW. In some cases, the improvement obtained by AdaBoost-LightGBM over RF was only marginal, and in Moga the reported RF metrics were slightly stronger than those of the best AdaBoost-LightGBM configuration. This suggests that boosting added value mainly under conditions where the standalone RF still left systematic residual structure, whereas in more stable or strongly autoregressive cases the base RF was already sufficient to reproduce most of the available signal. Based on the statistical metrics, SSWM9–RF achieved the lowest errors, with RMSE = 0.07, nRMSE = 0.23, RSR = 0.65, and MAE = 0.06. In terms of model accuracy, two statistical indices have been used, such as NSE 0.58 and CC 0.76, respectively, as shown in Table 6. Thus, SSWM9 appears to be the most suitable configuration for predicting SSW in Moga. Its final form is given in Eq. (9).

9 $$\begin{gathered} f = SSW\left( {t - 1} \right),~SSW\left( {t - 2} \right),~SSW\left( {t - 3} \right),~SSW\left( {t - 4} \right),~SSW\left( {t - 5} \right),SSW\left( {t - 6} \right), \hfill \\ SSW\left( {t - 7} \right),SSW\left( {t - 8} \right),SSW\left( {t - 9} \right),~SSW\left( {t - 10} \right),~SSW\left( {t - 11} \right),~SSW\left( {t - 9} \right) \hfill \\ \end{gathered}$$

A direct comparison with AdaBoost-LightGBM suggests that the benefit of boosting should be interpreted cautiously. Because standalone RF was already the strongest bagging-based learner, the gain from AdaBoost was generally modest and appears to be most useful where residual bias or difficult wetness transitions remained insufficiently captured by RF alone.

The same model parameters were also used for SSW prediction in Hoshiarpur (Fig. 8). In Hoshiarpur, the SSWM11–XGBoost model produced higher NSE and CC values and lower error metrics compared to the other bagging-based models (Fig. 9). The SSWM11– XGBoost model achieved CC = 0.82, NSE = 0.66, RMSE = 0.08, nRMSE = 0.23, RSR = 0.58, and MAE = 0.06 (Fig. 10). The remaining bagging-based models yielded lower performance metrics, as summarized in Table 6. The corresponding bagging-based model for Hoshiarpur is given in Eq. (10).

**Table 6:** Results of SSW of proposed models during testing phase.

Moga location																				
SSWM1											SSWM2									
Statistical Indices	RF	XGBoost	Light GBM	LMT	CART	AdaBoost-RF	AdaBoost-XGBoost	AdaBoost-Light GBM	AdaBoost-LMT	AdaBoost-CART	RF	XGBoost	Light GBM	LMT	CART	AdaBoost-RF	AdaBoost-XGBoost	AdaBoost-Light GBM	AdaBoost-LMT	AdaBoost-CART
RMSE	0.08	0.08	0.09	0.09	0.13	0.09	0.1	0.09	0.09	0.10	0.07	0.08	0.07	0.09	0.09	0.09	0.1	0.08	0.11	0.08
nRMSE	0.27	0.27	0.29	0.25	0.31	0.3	0.34	0.29	0.27	0.27	0.24	0.02	0.24	0.25	0.25	0.28	0.32	0.26	0.30	0.22
RSR	0.77	0.75	0.77	0.84	0.96	0.81	0.95	0.8	0.89	0.91	0.67	0.68	0.67	0.83	0.84	0.78	0.89	0.74	1.00	0.73
MAE	0.07	0.07	0.07	0.02	0.06	0.07	0.8	0.07	0.03	0.02	0.06	0.06	0.06	0.03	0.03	0.07	0.08	0.06	0.04	0.01
NSE	0.4	0.43	0.41	0.29	0.08	0.33	0.08	0.34	0.21	0.17	0.53	0.53	0.54	0.31	0.29	0.4	0.19	0.45	0.00	0.47
CC	0.64	0.65	0.64	0.59	0.50	0.59	0.55	0.61	0.57	0.56	0.73	0.73	0.74	0.64	0.64	0.67	0.58	0.68	0.59	0.69
SSWM5											SSWM6									
Statistical Indices	RF	XGBoost	Light GBM	LMT	CART	AdaBoost-RF	AdaBoost-XGBoost	AdaBoost-Light GBM	AdaBoost-LMT	AdaBoost-CART	RF	XGBoost	Light GBM	LMT	CART	AdaBoost-RF	AdaBoost-XGBoost	AdaBoost-Light GBM	AdaBoost-LMT	AdaBoost-CART
RMSE	0.07	0.07	0.08	0.08	0.08	0.08	0.09	0.08	0.09	0.09	0.07	0.07	0.07	0.08	0.08	0.08	0.09	0.08	0.09	0.09
nRMSE	0.23	0.24	0.25	0.23	0.24	0.26	0.3	0.26	0.25	0.24	0.23	0.23	0.24	0.23	0.23	0.26	0.28	0.25	0.26	0.25
RSR	0.66	0.65	0.68	0.77	0.80	0.74	0.85	0.73	0.82	0.82	0.66	0.65	0.68	0.76	0.78	0.74	0.78	0.7	0.87	0.84
MAE	0.05	0.06	0.06	0.03	0.04	0.06	0.08	0.06	0.02	0.00	0.05	0.06	0.06	0.03	0.03	0.07	0.07	0.06	0.03	− 0.01
NSE	0.56	0.57	0.53	0.41	0.36	0.45	0.28	0.46	0.33	0.33	0.57	0.57	0.53	0.42	0.40	0.45	0.38	0.5	0.24	0.29
CC	0.75	0.75	0.73	0.71	0.69	0.69	0.6	0.68	0.63	0.63	0.75	0.76	0.74	0.71	0.70	0.69	0.67	0.72	0.60	0.66
SSWM9											SSWM11									
Statistical Indices	RF	XGBoost	Light GBM	LMT	CART	AdaBoost-RF	AdaBoost-XGBoost	AdaBoost-Light GBM	AdaBoost-LMT	AdaBoost-CART	RF	XGBoost	Light GBM	LMT	CART	AdaBoost-RF	AdaBoost-XGBoost	AdaBoost-Light GBM	AdaBoost-LMT	AdaBoost-CART
RMSE	0.07	0.09	0.07	0.09	0.09	0.07	0.09	0.07	0.09	0.09	0.07	0.07	0.07	0.08	0.09	0.08	0.08	0.08	0.08	0.08
nRMSE	0.23	0.28	0.24	0.25	0.27	0.24	0.3	0.23	0.25	0.26	0.24	0.23	0.24	0.24	0.25	0.27	0.28	0.26	0.23	0.24
RSR	0.65	0.77	0.67	0.82	0.90	0.67	0.84	0.65	0.83	0.87	0.66	0.65	0.67	0.80	0.84	0.75	0.77	0.74	0.77	0.79
MAE	0.06	0.07	0.06	0.31	0.30	0.06	0.07	0.06	0.30	0.30	0.06	0.06	0.06	0.30	0.30	0.07	0.07	0.06	0.30	0.30
NSE	0.58	0.4	0.55	0.33	0.19	0.54	0.29	0.57	0.32	0.25	0.56	0.57	0.55	0.36	0.30	0.43	0.4	0.45	0.40	0.38
CC	0.76	0.64	0.74	0.67	0.65	0.76	0.63	0.76	0.70	0.61	0.75	0.76	0.74	0.72	0.70	0.68	0.69	0.69	0.74	0.67
SSWM12																				
Statistical Indices	RF	XGBoost	Light GBM	LMT	CART	AdaBoost-RF	AdaBoost-XGBoost	AdaBoost-Light GBM	AdaBoost-LMT	AdaBoost-CART										
RMSE	0.07	0.09	0.07	0.08	0.09	0.09	0.10	0.08	0.08	0.08										
nRMSE	0.24	0.28	0.24	0.23	0.25	0.028	0.31	0.25	0.24	0.24										
RSR	0.68	0.77	0.67	0.76	0.84	0.77	0.86	0.71	0.80	0.81										
MAE	0.06	0.07	0.06	0.30	0.30	0.07	0.08	0.06	0.30	0.30										
NSE	0.53	0.4	0.55	0.43	0.30	0.4	0.25	0.50	0.36	0.35										
CC	0.73	0.66	0.74	0.75	0.68	0.67	0.6	0.72	0.71	0.66										
Hoshiarpur location																				
SSWM1											SSWM2									
Statistical Indices	RF	XGBoost	Light GBM	LMT	CART	AdaBoost-RF	AdaBoost-XGBoost	AdaBoost-Light GBM	AdaBoost-LMT	AdaBoost-CART	RF	XGBoost	Light GBM	LMT	CART	AdaBoost-RF	AdaBoost-XGBoost	AdaBoost-Light GBM	AdaBoost-LMT	AdaBoost-CART
RMSE	0.1	0.1	0.1	0.11	0.13	0.1	0.12	0.1	0.12	0.12	0.09	0.09	0.09	0.11	0.14	0.1	0.115	0.1	0.11	0.11
nRMSE	0.28	0.29	0.29	0.26	0.31	0.3	0.34	0.3	0.27	0.28	0.25	0.25	0.26	0.26	0.31	0.29	0.33	0.29	0.25	0.25
RSR	0.71	0.73	0.72	0.80	0.96	0.75	0.86	0.75	0.83	0.85	0.63	0.62	0.65	0.81	0.97	0.74	0.83	0.73	0.77	0.78
MAE	0.8	0.08	0.08	0.04	0.06	0.08	0.09	0.08	0.04	0.04	0.07	0.07	0.07	0.05	0.06	0.08	0.09	0.08	0.05	0.05
NSE	0.5	0.46	0.48	0.37	0.08	0.43	0.25	0.43	0.31	0.27	0.6	0.62	0.58	0.35	0.06	0.45	0.3	0.46	0.41	0.39
CC	0.72	0.70	0.71	0.67	0.50	0.69	0.6	0.68	0.63	0.61	0.78	0.79	0.77	0.74	0.51	0.68	0.64	0.72	0.72	0.72
SSWM5											SSWM6									
Statistical Indices	RF	XGBoost	Light GBM	LMT	CART	AdaBoost-RF	AdaBoost-XGBoost	AdaBoost-Light GBM	AdaBoost-LMT	AdaBoost-CART	RF	XGBoost	Light GBM	LMT	CART	AdaBoost-RF	AdaBoost-XGBoost	AdaBoost-Light GBM	AdaBoost-LMT	AdaBoost-CART
RMSE	0.08	0.09	0.09	0.11	0.11	0.09	0.12	0.1	0.11	0.11	0.09	0.09	0.09	0.11	0.11	0.09	0.11	0.09	0.11	0.11
nRMSE	0.25	0.27	0.26	0.24	0.26	0.27	0.33	0.27	0.25	0.26	0.25	0.27	0.27	0.25	0.27	0.27	0.31	0.26	0.25	0.26
RSR	0.62	0.07	0.66	0.76	0.82	0.69	0.84	0.69	0.76	0.81	0.62	0.67	0.67	0.77	0.82	0.69	0.78	0.65	0.76	0.81
MAE	0.06	0.07	0.07	0.05	0.06	0.07	0.09	0.7	0.05	0.06	0.07	0.07	0.07	0.06	0.06	0.07	0.08	0.06	0.06	0.06
NSE	0.61	0.55	0.56	0.43	0.33	0.52	0.29	0.52	0.42	0.35	0.61	0.55	0.55	0.41	0.32	0.52	0.38	0.58	0.42	0.35
CC	0.79	0.76	0.76	0.78	0.71	0.74	0.62	0.73	0.76	0.73	0.79	0.76	0.75	0.78	0.71	0.73	0.66	0.77	0.76	0.74
SSWM9											SSWM11									
Statistical Indices	RF	XGBoost	Light GBM	LMT	CART	AdaBoost-RF	AdaBoost-XGBoost	AdaBoost-Light GBM	AdaBoost-LMT	AdaBoost-CART	RF	XGBoost	Light GBM	LMT	CART	AdaBoost-RF	AdaBoost-XGBoost	AdaBoost-Light GBM	AdaBoost-LMT	AdaBoost-CART
RMSE	0.09	0.09	0.09	0.12	0.12	0.1	0.13	0.1	0.12	0.12	0.08	0.08	0.09	0.10	0.13	0.1	0.11	0.09	0.11	0.12
nRMSE	0.25	0.25	0.26	0.27	0.29	0.29	0.37	0.29	0.27	0.28	0.24	0.23	0.25	0.24	0.29	0.28	0.32	0.26	0.25	0.27
RSR	0.63	0.64	0.64	0.85	0.89	0.73	0.93	0.75	0.83	0.86	0.6	0.58	0.63	0.75	0.90	0.7	0.796	0.64	0.77	0.83
MAE	0.07	0.07	0.07	0.07	0.06	0.08	0.1	0.08	0.07	0.07	0.06	0.06	0.07	0.06	0.06	0.08	0.08	0.07	0.06	0.07
NSE	0.6	0.59	0.59	0.28	0.21	0.47	0.13	0.46	0.31	0.26	0.63	0.66	0.6	0.44	0.19	0.5	0.36	0.58	0.41	0.30
CC	0.78	0.78	0.77	0.78	0.63	0.7	0.56	0.7	0.76	0.75	0.80	0.82	0.78	0.82	0.62	0.74	0.68	0.77	0.81	0.78
SSWM12																				
Statistical Indices	RF	XGBoost	Light GBM	LMT	CART	AdaBoost-RF	AdaBoost-XGBoost	AdaBoost-Light GBM	AdaBoost-LMT	AdaBoost-CART										
RMSE	0.09	0.09	0.09	0.11	0.13	0.09	0.1	0.09	0.11	0.11										
nRMSE	0.25	0.27	0.26	0.25	0.29	0.26	0.29	0.26	0.25	0.26										
RSR	0.62	0.68	0.64	0.77	0.90	0.65	0.74	0.65	0.77	0.80										
MAE	0.07	0.07	0.07	0.06	0.06	0.07	0.08	0.07	0.06	0.06										
NSE	0.62	0.54	0.59	0.41	0.19	0.57	0.45	0.58	**0.41**	0.37										
CC	0.80	0.74	0.77	0.81	0.61	0.77	0.73	0.77	**0.82**	0.81										
Firozpur location																				
SSWM1											SSWM2									
Statistical Indices	RF	XGBoost	Light GBM	LMT	CART	AdaBoost-RF	AdaBoost-XGBoost	AdaBoost-Light GBM	AdaBoost-LMT	AdaBoost-CART	RF	XGBoost	Light GBM	LMT	CART	AdaBoost-RF	AdaBoost-XGBoost	AdaBoost-Light GBM	AdaBoost-LMT	AdaBoost-CART
RMSE	0.1	0.09	0.1	0.10	0.11	0.11	0.12	0.1	0.10	0.11	0.1	0.09	0.1	0.1	0.10	0.1	0.11	0.1	0.10	0.10
nRMSE	0.37	0.35	0.36	0.31	0.33	0.4	0.42	0.36	0.32	0.32	0.37	0.36	0.36	0.29	0.31	0.37	0.41	0.36	0.31	0.31
RSR	0.9	0.88	0.88	0.88	0.96	0.99	1.03	0.89	0.91	0.92	0.91	0.88	0.88	0.83	0.88	0.91	1	0.88	0.89	0.89
MAE	0.08	0.08	0.08	0.02	0.03	0.09	0.1	0.08	0.03	0.03	0.08	0.08	0.08	0.03	0.03	0.08	0.09	0.08	0.03	0.03
NSE	0.18	0.22	0.21	0.22	0.08	− 0.004	− 0.08	0.21	0.18	0.16	0.17	0.22	0.22	0.31	0.22	0.16	− 0.02	0.21	0.21	0.22
CC	0.49	0.51	0.5	0.54	0.41	0.37	0.4	0.49	0.49	0.48	0.48	0.51	0.5	0.63	0.54	0.45	0.36	0.52	0.52	0.53
SSWM5											SSWM6									
Statistical Indices	RF	XGBoost	Light GBM	LMT	CART	AdaBoost-RF	AdaBoost-XGBoost	AdaBoost-Light GBM	AdaBoost-LMT	AdaBoost-CART	RF	XGBoost	Light GBM	LMT	CART	AdaBoost-RF	AdaBoost-XGBoost	AdaBoost-Light GBM	AdaBoost-LMT	AdaBoost-CART
RMSE	0.1	0.1	0.1	0.09	0.10	0.11	0.12	0.1	0.10	0.10	0.1	0.09	0.10	0.12	0.10	0.11	0.12	0.1	0.10	0.10
nRMSE	0.36	0.36	0.36	0.28	0.31	0.39	0.42	0.38	0.31	0.30	0.36	0.34	0.35	0.37	0.31	0.39	0.43	0.38	0.31	0.30
RSR	0.89	0.89	0.88	0.81	0.88	0.97	1	0.93	0.90	0.87	0.88	0.85	0.86	1.07	0.88	0.96	1	0.94	0.90	0.88
MAE	0.08	0.08	0.08	0.03	0.03	0.09	0.09	0.09	0.04	0.03	0.07	0.08	0.07	0.02	0.03	0.09	0.1	0.08	0.03	0.03
NSE	0.19	0.2	0.21	0.34	0.23	0.05	− 0.085	0.13	0.19	0.25	0.21	0.23	0.26	− 0.14	0.23	0.07	− 0.16	0.11	0.18	0.23
CC	0.48	0.49	0.49	0.63	0.55	0.39	0.39	0.42	0.55	0.58	0.5	0.55	0.54	0.41	0.55	0.47	0.43	0.47	0.53	0.56
SSWM9											SSWM11									
Statistical Indices	RF	XGBoost	Light GBM	LMT	CART	AdaBoost-RF	AdaBoost-XGBoost	AdaBoost-Light GBM	AdaBoost-LMT	AdaBoost-CART	RF	XGBoost	Light GBM	LMT	CART	AdaBoost-RF	AdaBoost-XGBoost	AdaBoost-Light GBM	AdaBoost-LMT	AdaBoost-CART
RMSE	0.09	0.1	0.1	0.10	0.11	0.11	0.12	0.1	0.10	0.10	0.09	0.1	0.09	0.09	0.11	0.1	0.12	0.1	0.10	0.10
nRMSE	0.34	0.35	0.34	0.29	0.34	0.4	0.43	0.36	0.31	0.31	0.32	0.36	0.33	0.28	0.32	0.37	0.42	0.36	0.31	0.30
RSR	0.84	0.87	0.85	0.84	0.97	0.99	1	0.9	0.88	0.89	0.8	0.9	0.83	0.81	0.93	0.91	1	0.89	0.88	0.88
MAE	0.07	0.08	0.07	0.03	0.04	0.09	0.09	0.08	0.04	0.04	0.07	0.08	0.07	0.03	0.03	0.07	0.09	0.07	0.04	0.04
NSE	0.28	0.24	0.27	0.30	0.07	0.37	− 0.134	0.18	0.23	0.20	0.35	0.19	0.31	0.34	0.13	0.17	− 0.06	0.2	0.22	0.23
CC	0.56	0.52	0.54	0.62	0.44	0.004	0.36	0.48	0.60	0.57	0.62	0.56	0.58	0.64	0.47	0.5	0.46	0.5	0.60	0.60
SSWM12																				
Statistical Indices	RF	XGBoost	Light GBM	LMT	CART	AdaBoost-RF	AdaBoost-XGBoost	AdaBoost-Light GBM	AdaBoost-LMT	AdaBoost-CART										
RMSE	0.09	0.10	0.10	0.10	0.11	0.1	0.11	0.1	0.11	0.10										
nRMSE	0.33	0.34	0.34	0.30	0.33	0.36	0.38	0.36	0.32	0.32										
RSR	0.82	0.84	0.84	0.86	0.94	0.88	0.95	0.88	0.93	0.91										
MAE	0.08	0.07	0.08	0.03	0.03	0.07	0.08	0.07	0.04	0.02										
NSE	0.32	0.29	0.28	0.26	0.11	0.23	0.09	0.22	0.14	0.17										
CC	0.59	0.57	0.56	0.59	0.44	0.53	0.49	0.53	0.52	0.56										

*SSWM* Soil surface wetness model.

10 $$\begin{gathered} f = SSW\left( {t - 1} \right),~SSW\left( {t - 2} \right),~SSW\left( {t - 3} \right),~SSW\left( {t - 4} \right),~SSW\left( {t - 5} \right),SSW\left( {t - 6} \right), \hfill \\ SSW\left( {t - 7} \right),SSW\left( {t - 8} \right),SSW\left( {t - 9} \right),~SSW\left( {t - 10} \right),~SSW\left( {t - 11} \right),~SSW\left( {t - 9} \right) \hfill \\ \end{gathered}$$

Following the analysis of Moga and Hoshiarpur, the Firozpur results were examined using statistical indices and visual plots. Based on the statistical results, the LMT configuration provided the highest accuracy among the bagging-based models in Firozpur. Accordingly, the recommended SSW model for Firozpur is presented in Eq. (11).

11 $$\begin{gathered} f = SSW\left( {t - 1} \right),~SSW\left( {t - 2} \right),~SSW\left( {t - 3} \right),~SSW\left( {t - 4} \right),~SSW\left( {t - 5} \right),SSW\left( {t - 6} \right), \hfill \\ SSW\left( {t - 7} \right),SSW\left( {t - 8} \right),SSW\left( {t - 9} \right),~SSW\left( {t - 10} \right),~SSW\left( {t - 11} \right) \hfill \\ \end{gathered}$$

In this proposed study area, the SSWM 11 (RF) model gives higher accuracy in terms of NSE & CC, and a lower value of error in the bagging-based models. It is demonstrated that the CC of the RF (SSWM 11) model is 0.62, NSE is 0.35, RMSE is 0.09, nRMSE is 0.32, RSR is 0.80, and MAE is 0.07, respectively. Overall, the machine learning models effectively predicted monthly SSW across the study sites under various input–output combinations.

Although RMSE and MAE provide absolute error magnitudes, their practical significance depends on the range and variability of soil surface wetness (SSW). Therefore, error interpretation is supported by normalized metrics (nRMSE and RSR) reported in Table 6, which express prediction errors relative to the mean and standard deviation of the reference series, enabling comparison across locations. A negative NSE indicates that the model predictions are less accurate than a simple mean-based benchmark, implying that the model fails to capture the temporal variability of SSW adequately for that particular case. In addition, the predicted versus reference plots in Fig. 8 visually illustrate model bias and dispersion during the testing period. Scenarios characterized by higher nRMSE/RSR values and increased scatter in Fig. 8 correspond to lower or negative NSE, indicating limited skill in reproducing absolute SSW magnitudes, whereas tighter clustering around the 1:1 line reflects improved relative performance.

### Discussion of proposed models

The primary motivation of the present study was to predict SSW using various input-combination modeling techniques across multiple locations. To achieve this, several hybrid machine learning (ML) models were developed, incorporating the AdaBoost algorithm for hybrid construction. The performance of the proposed model is illustrated using time series plot (TSP), scatter plots, radar diagrams, and taylor diagram (TD) to provide a clear visual understanding of the results.

#### Discussion of bagging-based models

In this study, several diagrams are plotted for bagging-based models to provide a better understanding of the observed and predicted SSW. Bagging models reduce variance by averaging multiple bootstrap samples, but they typically do not improve model bias. In the present study, the bagging-based models showed comparatively weaker performance under the examined lag-based surrogate modeling framework; however, this should be interpreted as a dataset- and context-specific finding rather than as a general limitation of bagging for nonlinear hydrological systems. This explains the relatively weaker performance in areas with higher climatic variability such as Hoshiarpur. The TSP for bagging-based model in the proposed study areas shows a good agreement between observed and predicted SSW values. These plots help in clearly understanding the SSW conditions of the study area, and they also enable the estimation of approximate SSW conditions in other areas with similar hydro-meteorological and hydro-geological characteristics. For the SSWM 9 model, both the time series and scatter plots indicate good agreement between observed and modeled values in Moga as shown in Fig. 4. Each scatter diagram includes a line of perfect fit (LPF), which highlights the better performance of the model in predicting SSW.

**Fig. 5:**
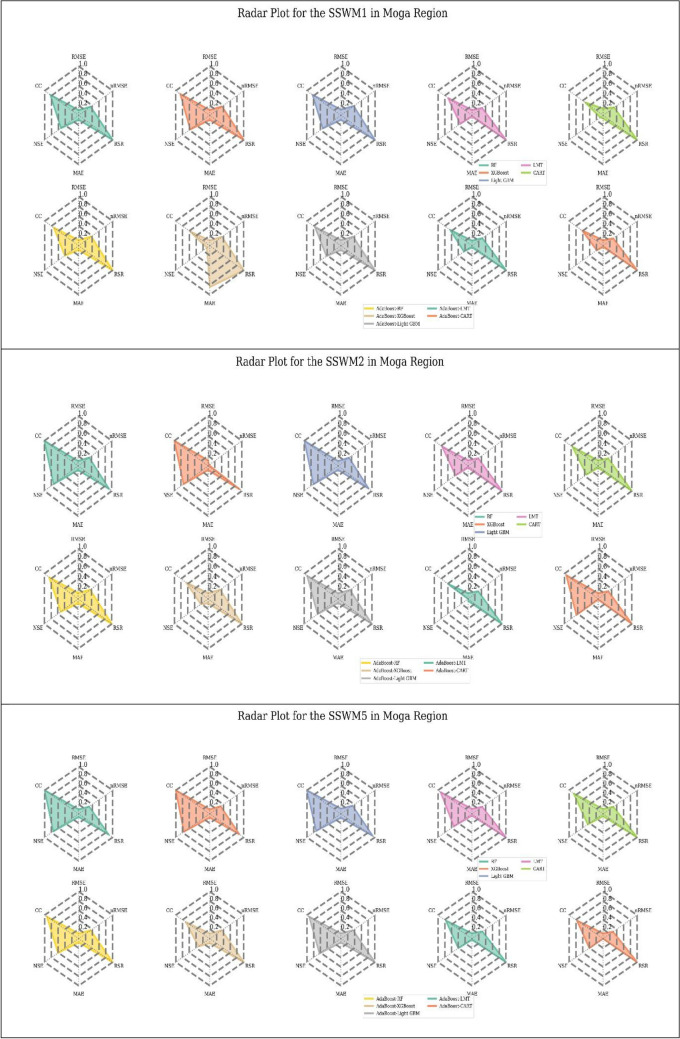

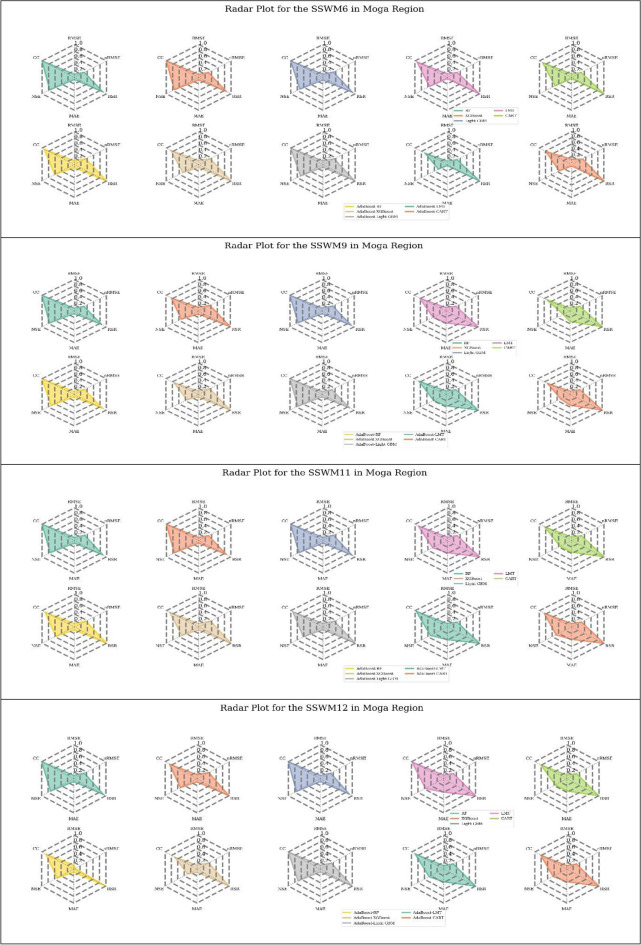
Radar diagram showing the relationship between statistical indices along the proposed predicted SSW of the Moga region during testing of the models.

**Fig. 6:**
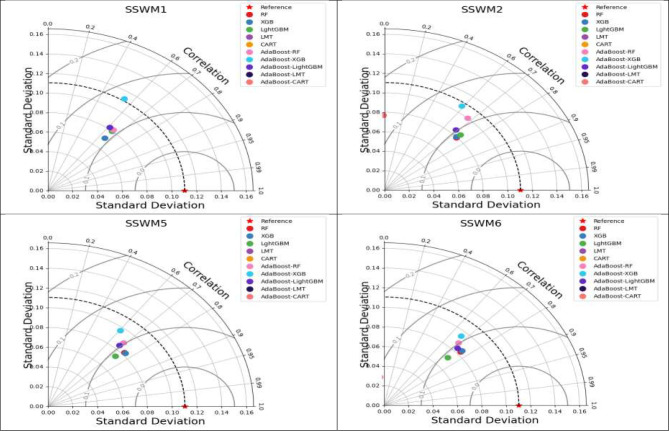
Taylor diagram showing the relationship of correlation coefficient between actual and predicted SSW of the Moga region during testing of the models.

**Fig. 7:**
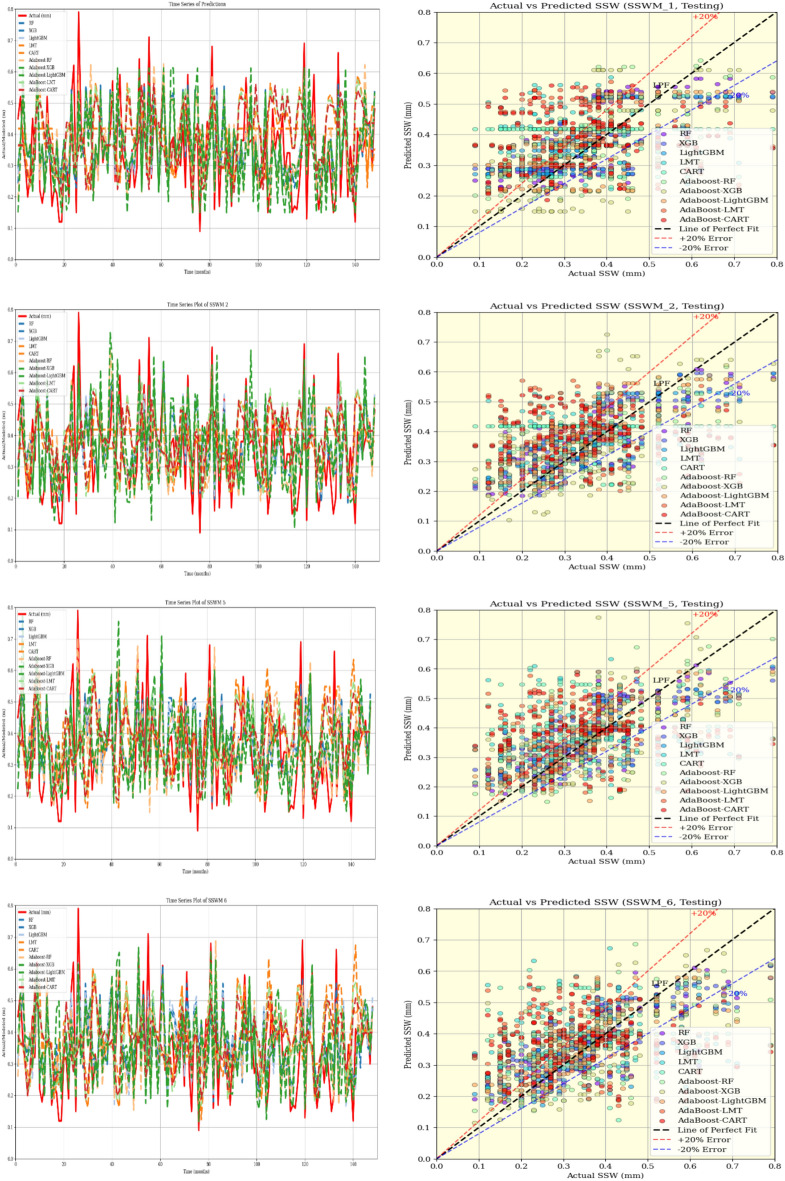

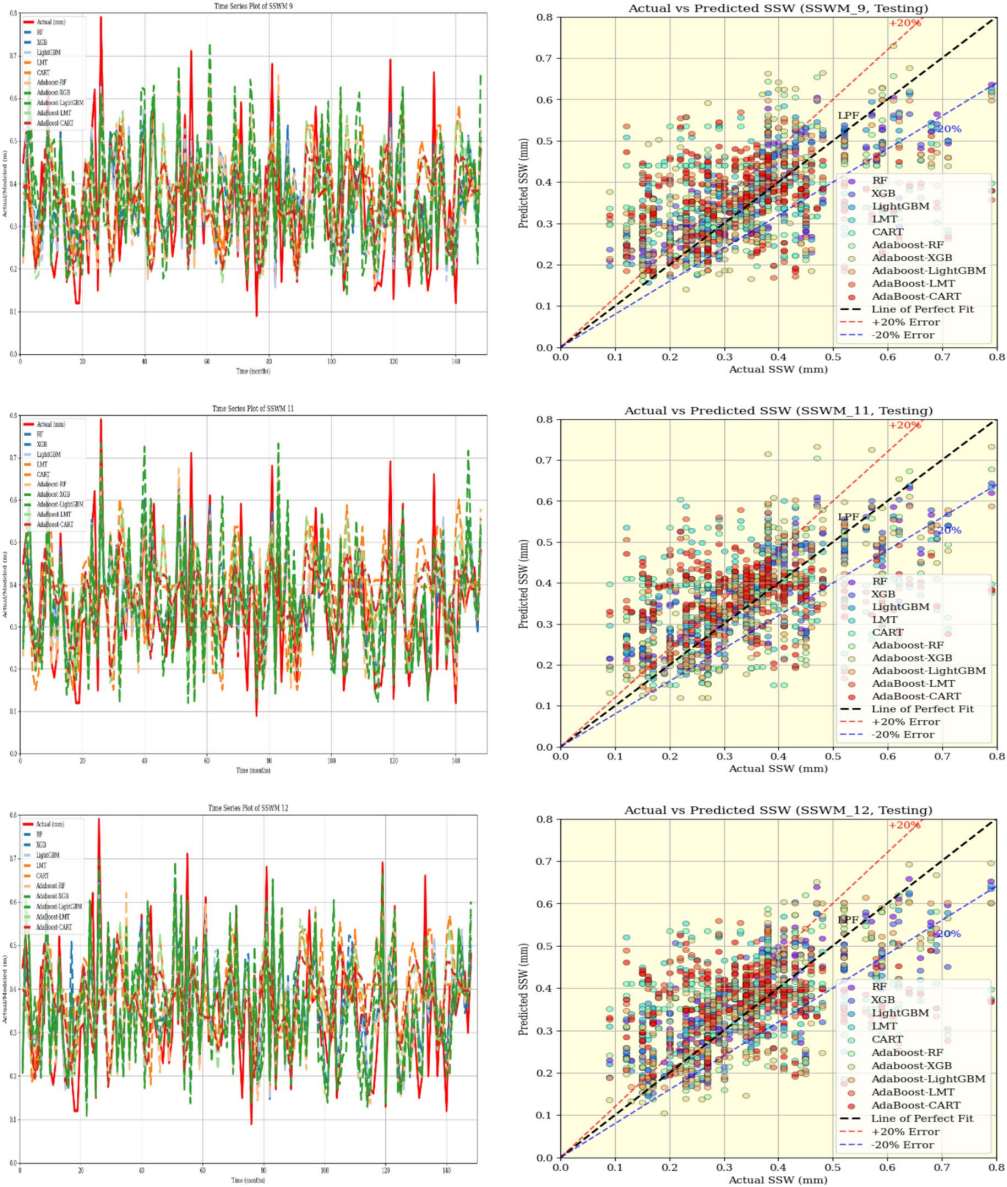
Time series plot of observed and predicted models and scatter plot between actual and predicted SSW of the Hoshiarpur during testing of the models.

**Fig. 8:**
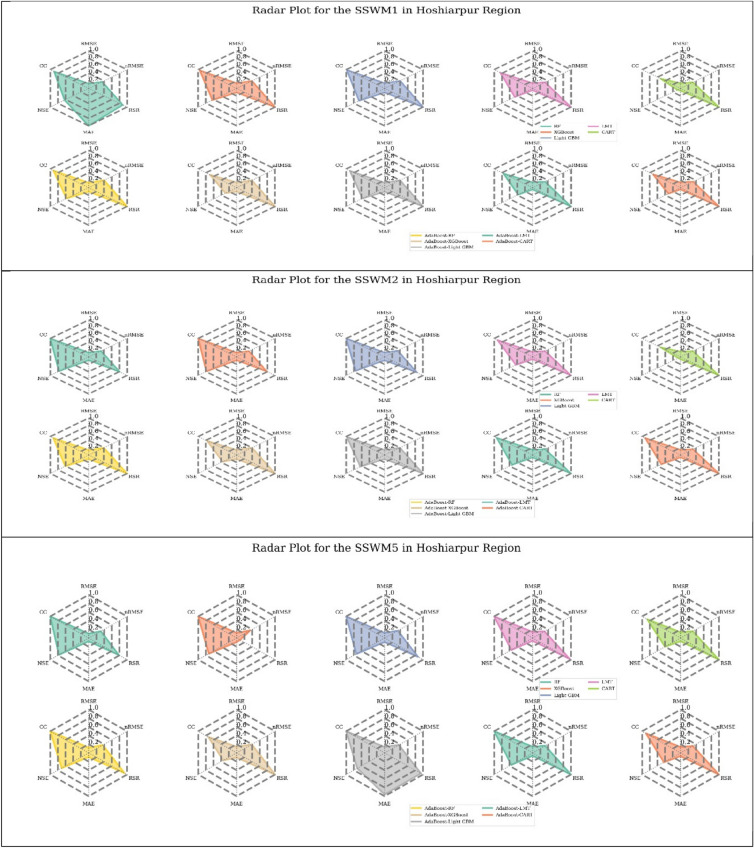

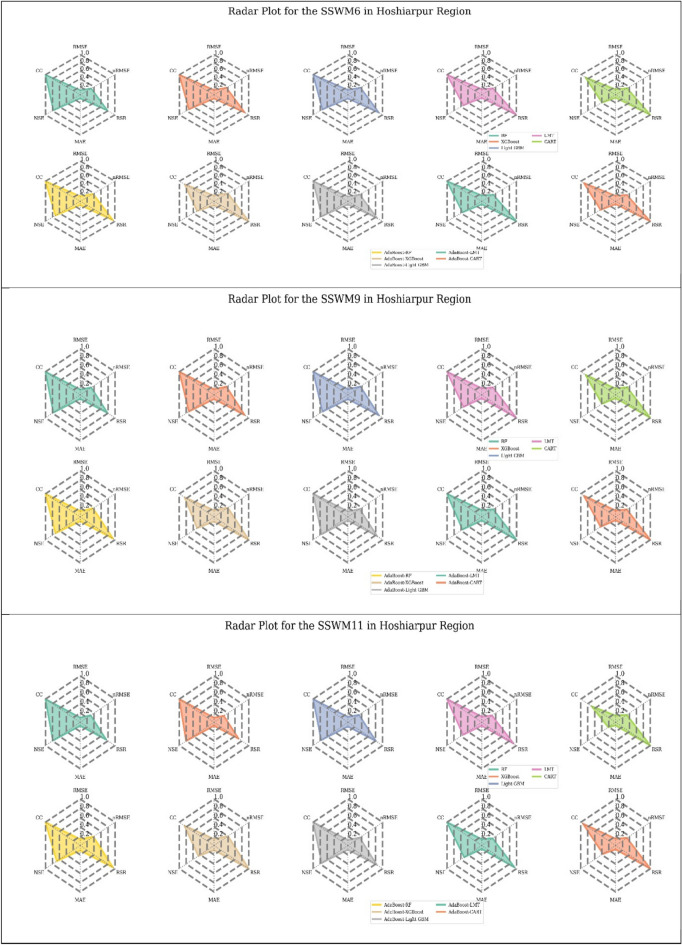

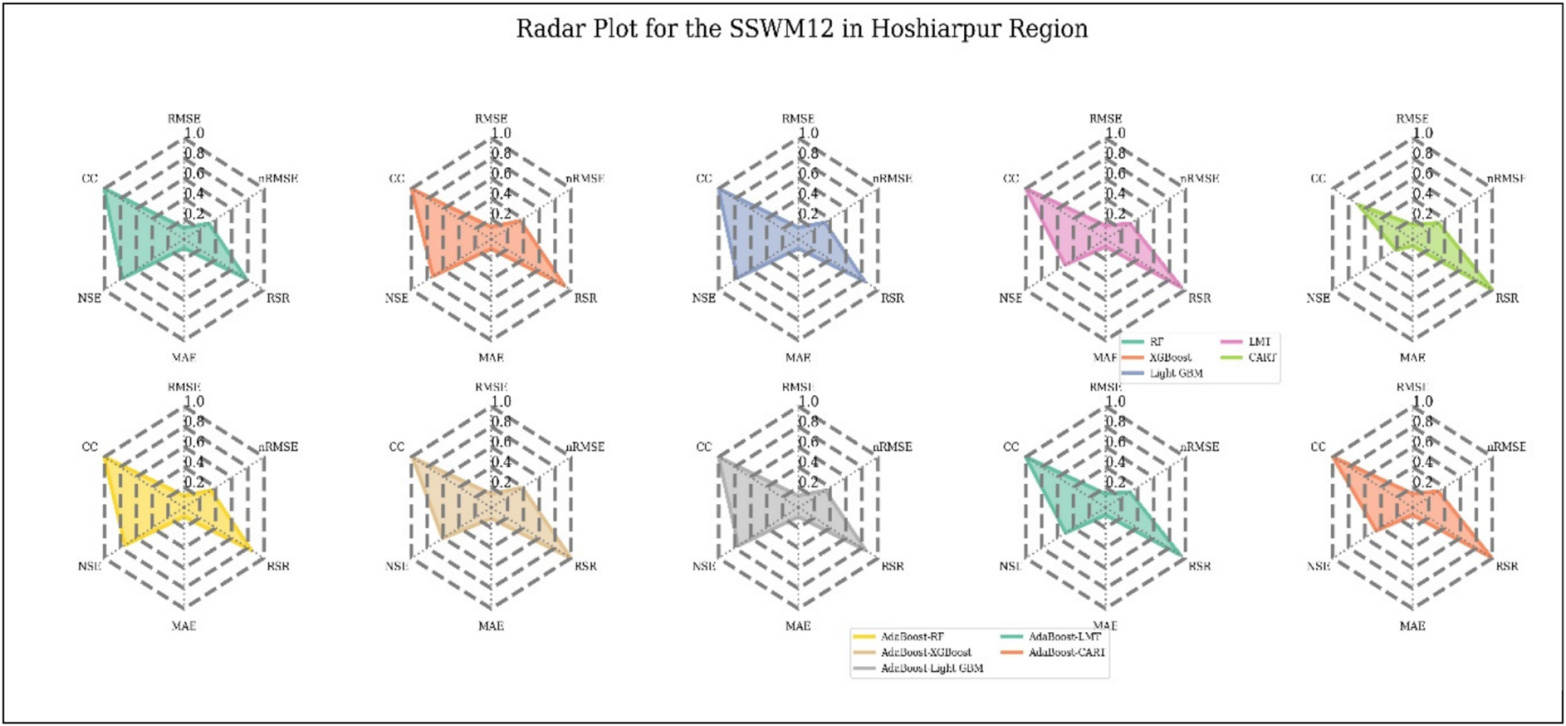
Radar diagram showing the relationship between statistical indices along the proposed models of SSW in Hoshiarpur during testing of the models.

**Fig. 9:**
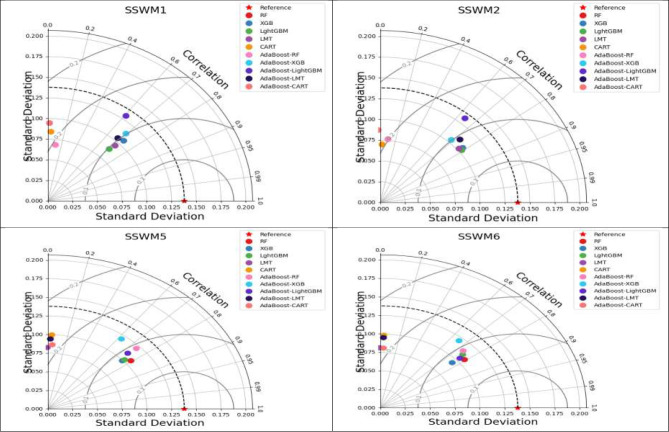
Taylor diagram showing the relationship of correlation coefficient between observed and predicted models of SSW in Hoshiarpur during testing of the models.

For visualizing model performance, a radar diagram has also been presented for the Moga area. The radar diagram provides a multi-criteria comparison of statistical indices extracted from the bagging-based model (SSWM 9) as depicted in Fig. 5. Furthermore, the TD serves as a vital tool for the visualization and interpretation of model statistics, including the standard deviation (SD) and correlation coefficient (CC). The TD provides individual values of SD and CC for the bagging-based model (SSWM 9) in the Moga area, as shown in Fig. 6. Similarly, the SSWM 11 model provides better results for the Hoshiarpur area, as shown in Figs. 7 and 8. The time-series plots provide clearer insights into SSW trends for researchers, academicians, policymakers, and water experts. To demonstrate the correlation between observed and predicted SSW results, a scatter plot for the Hoshiarpur area during the model testing period is presented in Fig. 9.

**Fig. 10:**
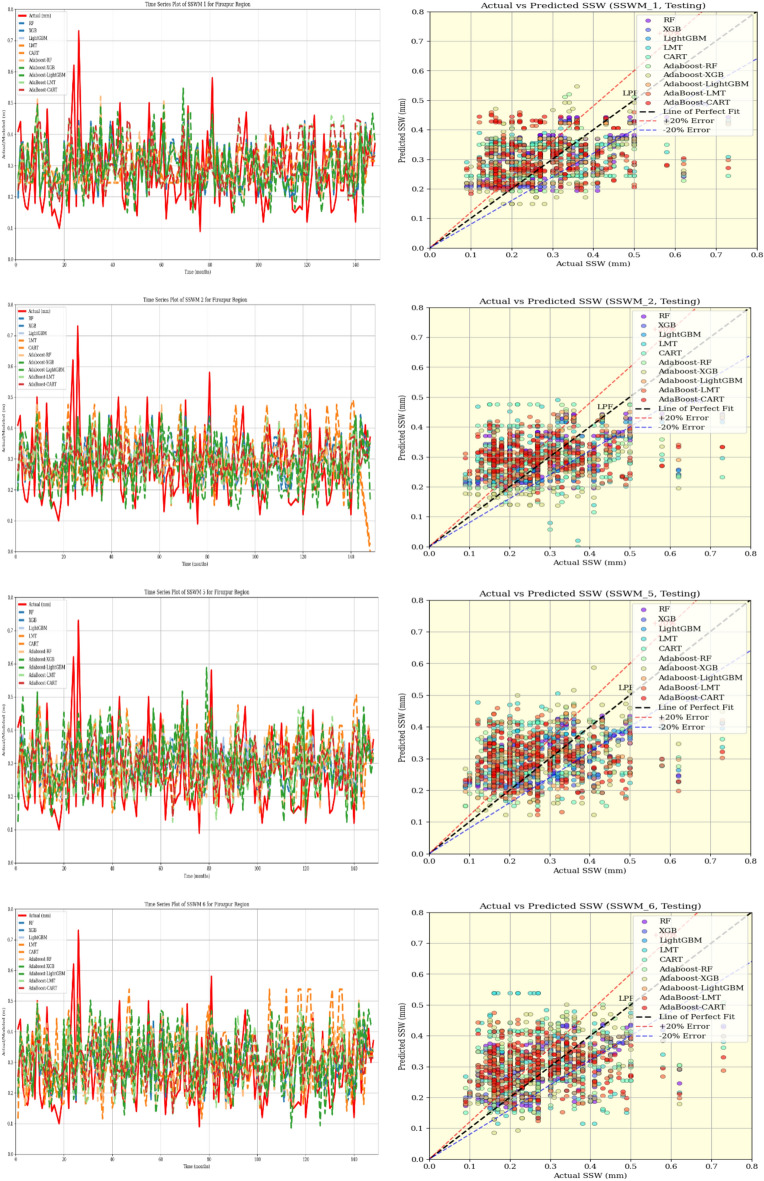

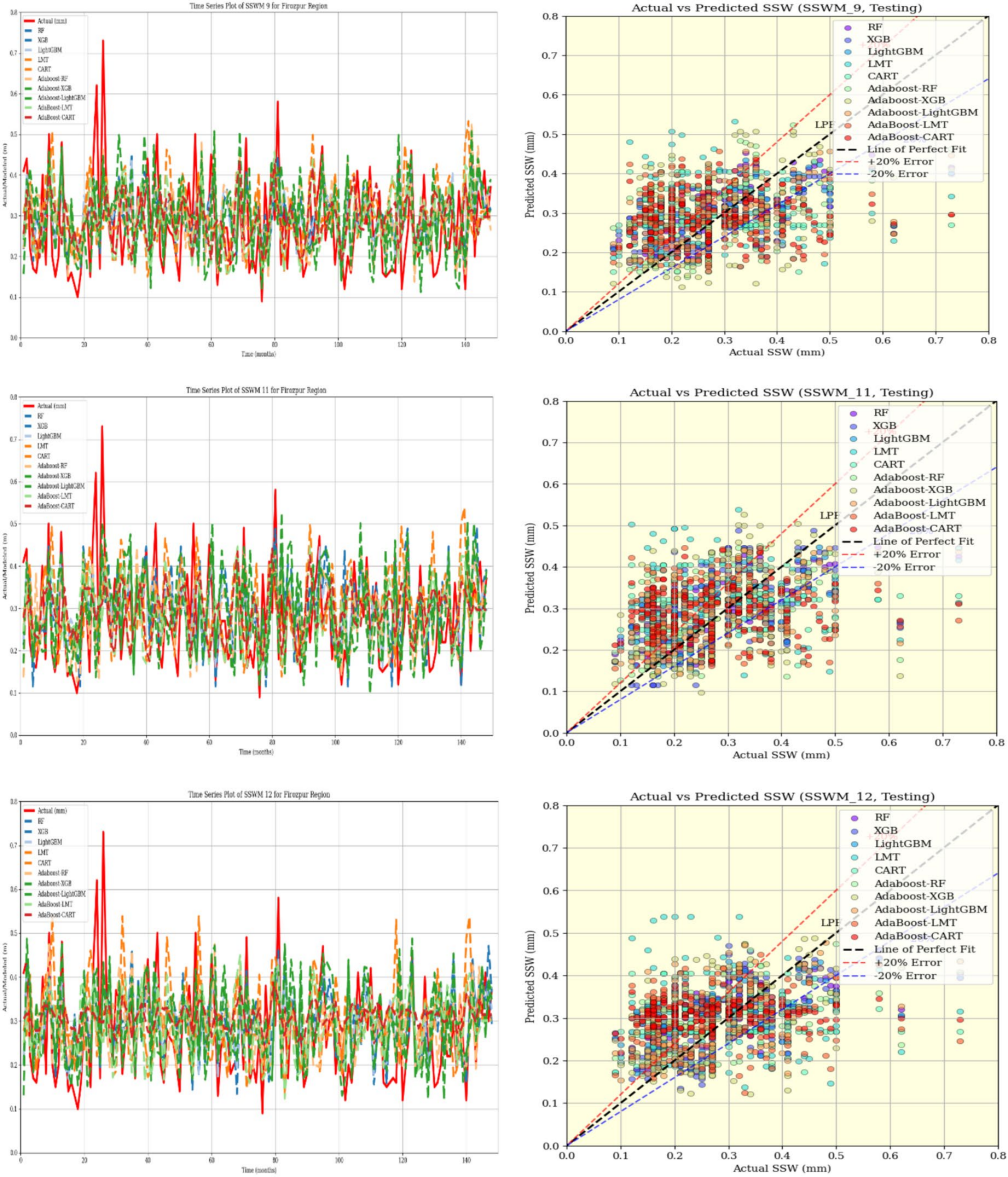
Time series plot of observed and predicted models and scatter plot between observed and predicted soil moisture wetness of the Firozpur during testing of the models.

**Fig. 11:**
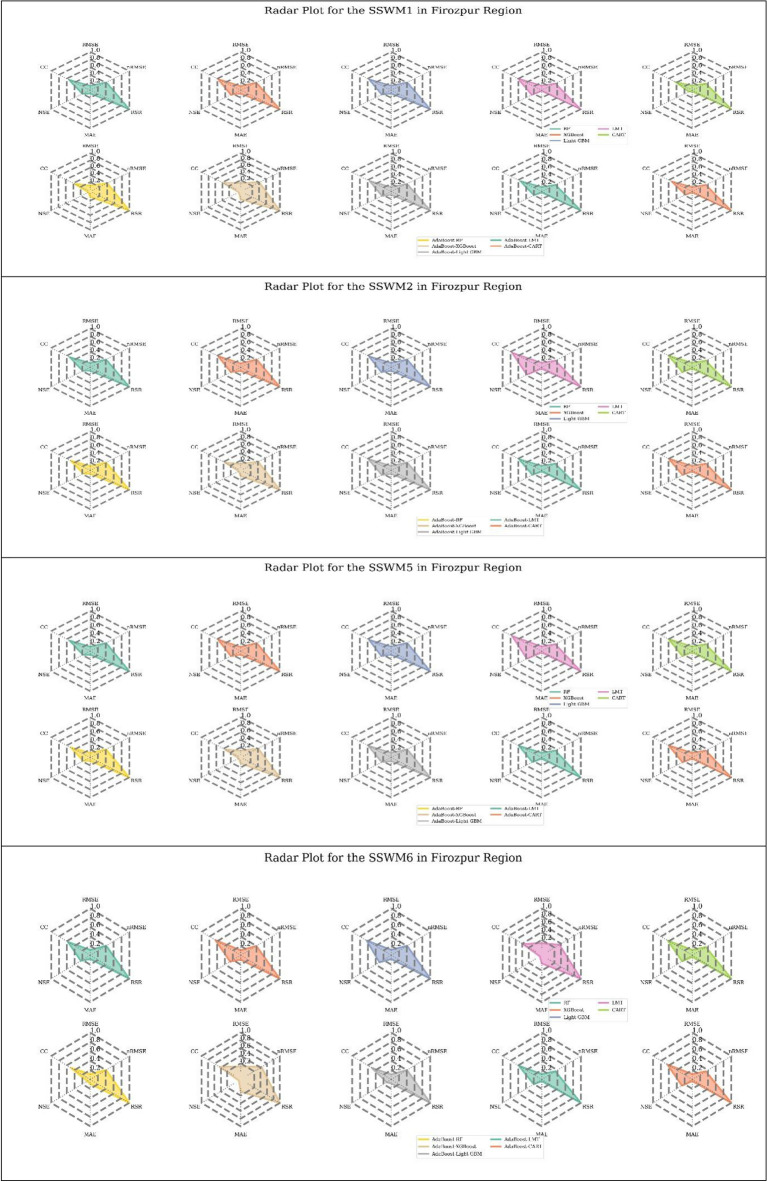

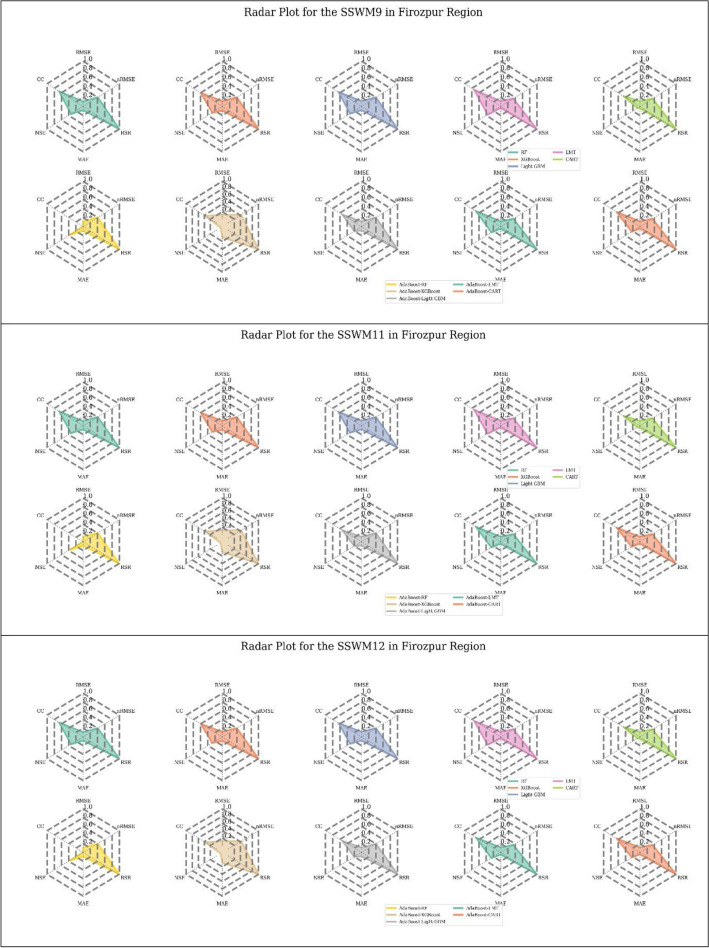
Radar diagram showing the relationship between statistical indices along the proposed models of SSW in Firopur during testing of the models.

**Fig. 12:**
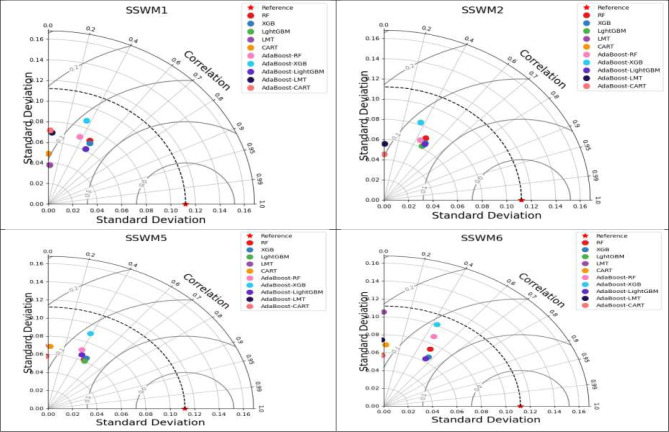
Taylor diagram showing the relationship of correlation coefficient between observed and predicted models of SSW in Firozpur during testing of the models.

Additionally, as discussed in the results section of SSWM 11 (XGBoost) model, it also provides satisfactory results for the Firozpur location as mentioned in Figs. 10 and 11. Similarly, TSP, scatter plot, radar diagram, and TD are plotted to enhance understanding of the model’s performance, as shown in Figs. 11 and 12. Overall, the bagging-based SSWM11–RF & XGBoost model performed better in Firozpur and Hoshiarpur compared to Moga. These spatial differences are hydrologically meaningful. Moga shows relatively uniform climatic conditions, allowing bagging models to generalize well. Hoshiarpur’s complex terrain, orographic rainfall, and stronger seasonal gradients likely contributed to conditions under which the bagging-based models performed less effectively in the present dataset^2^. Firozpur, characterized by high wind speeds and temperature-driven evaporation, exhibits more predictable moisture decay patterns, explaining why RF performs reasonably well there.

#### Discussion of hybrid models

The AdaBoost algorithm is more effective for predicting hydro-meteorological phenomena for better management of water resource management and crop planning. Boosting improves prediction by adaptively increasing the weight of misclassified or poorly predicted samples in each iteration. Since SSW dynamics often involve sharp transitions such as sudden wetting after rainfall or rapid drying under high evaporative demand this adaptive weighting enables AdaBoost to capture localized nonlinearities more effectively than bagging. With the help of the AdaBoost algorithm, we have developed four hybrid models for SSW prediction of the proposed study areas. The proposed hybrid models yielded more accurate results than the bagging-based models in the present study areas. This is supported by the statistical indices and visual comparisons (TSP, scatter plots, radar diagrams, and Taylor diagrams), all of which show strong agreement between observed and predicted values.

For Moga, the AdaBoost-LightBGM (SSWM9) model outperformed the other hybrid models. Therefore, the SSW distribution predicted by the AdaBoost-LightGBM model is clearly visible in the corresponding figures and tables. The superior performance of AdaBoost-LightGBM arises from its hybrid architecture. Decision-tree splits allow the model to distinguish SSW regimes driven by different hydro-meteorological conditions, while logistic regression functions at the terminal nodes capture smooth relationships within these regimes. This combination is well suited to SSW, which exhibits both threshold-dependent (rainfall-driven) and gradual (evaporation-driven) behaviour.

The proposed models are more appropriately interpreted as surrogate tools for comparative wetness assessment, screening-level analysis, and evaluation of relative temporal variability. Given the moderate-to-weak efficiency metrics in several cases, especially low or negative NSE values, they should not yet be considered validated for field-scale crop planning, precise irrigation scheduling, or other high-accuracy operational decisions. SSW information can also be used to estimate crop-water requirements, available water, wilting point, and field capacity. SSW in each location depends on several factors, including minimum and maximum temperature, rainfall, streamflow, wind velocity, sunshine hours, and evapotranspiration. The comparatively weaker performance observed at Firozpur suggests that the lag-based surrogate framework may not have fully captured the local controls influencing SSW dynamics at this site. Firozpur’s climatology is dominated by extreme temperatures, high vapour pressure deficit, and persistent dry winds, which accelerate surface evaporation. This explains why models consistently predict lower SSW in this area.

**Fig. 13:**
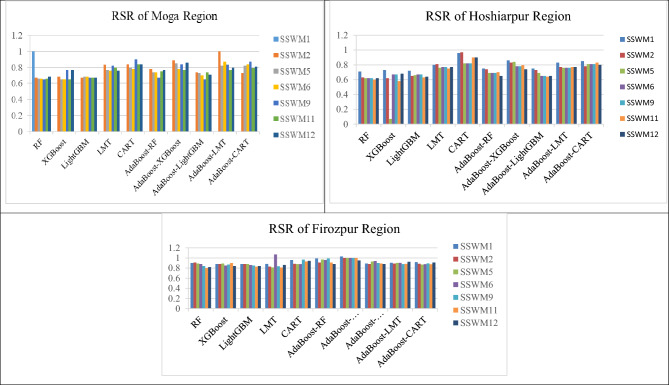
Bar chart representing the RSR of Moga, Hoshiarpur and Firozpur Regions.

These conditions have intensified due to the region’s growing population and rapid infrastructure development. The findings enhance understanding of the generalization and transferability of ML models across diverse geographical and environmental settings. The results demonstrated that the proposed hybrid model effectively predicts SSW using a combination of static and dynamic attributes^59^ of the Firozpur study area. A summary of the performance of individual models is also mentioned in Table 6 highlighting their initial identification and evaluation for the study area. A closer examination of the evaluation metrics (Table 6) indicates that the predictive skill of the tested models is moderate and, in some cases, weak, particularly when assessed using efficiency-based measures such as NSE and RSR. While correlation coefficients generally fall within the range of approximately 0.5–0.82, indicating moderate association, error-based metrics reveal notable limitations, including low NSE values and occasional negative NSE values, especially for the Firozpur location. These results suggest that correlation alone may overstate perceived model skill and must be interpreted alongside absolute and normalized error measures. Importantly, the superior performance of the AdaBoost–LightGBM model should be interpreted in a relative sense, as it consistently outperforms the other evaluated models but does not achieve uniformly better accuracy across all scenarios and sites. This behaviour reflects both the inherent difficulty of emulating modeled soil surface wetness dynamics and the constraints imposed by the autoregressive input structure and the use of GEOS-derived reference data. Consequently, the proposed models are best suited for screening-level analyses, comparative assessments, and applications where relative temporal variability and persistence are of primary interest, rather than for high-precision or operational soil moisture estimation. The Bar plot representing the RSR of Moga, Hoshiarpur and Firozpur regions as shown in Fig. 13.

The study’s findings enhance exploration into the generalization and transferability of ML models across several geographical and environmental settings. Future research will test models that include meteorological drivers (e.g., precipitation, temperature, radiation, wind) to improve interpretability and observational applicability. Furthermore, investigating the transferability of model parameters or architectures to other hydrological modeling tasks can foster knowledge exchange and facilitate the development of standardized approaches for addressing several water resources related challenges^1^. Model outputs may vary when input combinations, architectures, or random-seed selections are modified. Thus, in the future, it is of great importance to develop some robust and effective techniques and protocols for identifying the potential SSW characteristics, carefully determining reasonable input combinations and computational parameters^60,61^. From a hydrological perspective, accurate SSW prediction is crucial because surface wetness governs infiltration, soil evaporation, crop-water balance, and the onset of runoff. Therefore, the improved model performance demonstrated here can significantly enhance early-season irrigation planning, drought monitoring, and hydrological simulation accuracy in data-scarce regions.

The comparative analysis indicates that ensemble and hybrid approach generally outperform single tree-based learners in reproducing NASA POWER (GEOS-derived) soil surface wetness (SSW) dynamics; however, predictive skill is strongly location- and scenario-dependent and is not uniformly high. This is evident when CC is interpreted alongside RMSE/nRMSE and efficiency measures (NSE and RSR): several cases show only modest association (CC often < 0.6) with limited-to-moderate efficiency (low NSE and high RSR), and a few scenarios even yield negative NSE (e.g., Firozpur). Because NSE reflects a model’s ability to reproduce variability relative to the mean of the reference series, low or negative NSE indicates little improvement (or worse performance) compared to a mean-based benchmark for predicting absolute SSW values, particularly where temporal persistence is weak and short-term variability is high. From a practical standpoint, this limits the use of the models for high-accuracy operational applications requiring precise wetness magnitudes (e.g., field-scale irrigation scheduling), although moderate CC in some cases suggests the models can still capture relative temporal patterns and directional changes. Although CC indicates the strength of linear association between observed and predicted values, it does not by itself confirm satisfactory predictive skill; therefore, model performance was interpreted jointly using CC, NSE, and RSR. Accordingly, the results should be interpreted primarily as identifying the best-performing surrogate approach among those tested and delineating where such lag-based emulators are useful for screening/comparative analyses versus were incorporating explicit physical drivers (e.g., precipitation, evapotranspiration, and irrigation proxies) is necessary to improve efficiency and practical applicability.

The strong spatial variation in performance across Moga, Hoshiarpur, and Firozpur primarily reflects differences in the statistical structure of the target time series and the underlying hydro-climatic setting. Because the modeling framework is largely autoregressive (lagged SSW inputs), predictive skill depends strongly on (i) temporal persistence (autocorrelation/memory), (ii) strength and regularity of seasonality, and (iii) signal-to-noise ratio in the GEOS-derived SSW product. Locations with smoother seasonal cycles and stronger persistence are inherently more predictable with lag-only inputs, whereas locations with abrupt wetting–drying transitions and low persistence are difficult to emulate without explicit drivers. Hoshiarpur generally shows better performance because the wetness dynamics tend to be more seasonally structured and persistent, making lag-based models more effective. In contrast, poorer performance in Firozpur suggests weaker persistence and greater short-term variability in the modeled SSW signal, which reduces the information content of SSW(t − 1), SSW(t − 2), etc., and limits the achievable NSE even when CC is moderate. Differences among algorithms arise from how they manage nonlinearity, bias–variance trade-offs, and error distribution in noisy time series. Single tree-based models, particularly CART, are capable of capturing nonlinear thresholds, yet they remain sensitive to sampling variability and tree-structure complexity, which may result in overfitting or under fitting and hence unstable generalization. Ensemble approaches such as RF improve robustness through bootstrap aggregation, while gradient-boosting models such as XGBoost and LightGBM strengthen predictive accuracy by iteratively reducing residual error. Nevertheless, even these advanced learners may show limited performance when the target dynamics are strongly nonstationary or governed by abrupt changes.

Hybrid boosting models, particularly AdaBoost-LightGBM often showed strong relative performance because they combine two complementary strengths: (i) RF partitions the predictor space using a tree structure while fitting local models within terminal nodes, allowing smoother piecewise relationships than purely hard-split trees; and (ii) AdaBoost iteratively reweights difficult cases, which can reduce bias and improve fit to challenging segments of the time series. However, the comparative results indicate that this advantage is not uniform. Standalone RF was already a strong learner in many site–scenario combinations, which suggests that much of the predictive structure in the monthly GEOS-derived SSW series was already captured by the piecewise local model itself. Consequently, the added value of boosting was often modest and context dependent, with only marginal differences in several cases and occasional instances where standalone RF performed slightly better on individual metrics. This also highlights an important trade-off: AdaBoost-LightBGM introduces greater model complexity and can become more sensitive to noise or weakly structured signals. When the lag-based predictor structure already captures most of the available temporal information, boosting may provide only limited improvement, whereas under noisier or externally forced conditions it may increase the risk of overfitting and reduce generalizability from training to testing. Therefore, the role of AdaBoost in the present study should be interpreted as providing selective and incremental refinement rather than universal superiority over standalone RF.

Firozpur displays the weakest overall performance, including low and occasionally negative NSE, indicating that the modeled SSW time series contains variability that lag-based predictors cannot reliably anticipate. Several location-specific factors plausibly contribute: Firozpur lies in the south-western Punjab plains where rainfall is relatively lower and more episodic compared to sub-Himalayan/foothill-influenced zones. Combined with high temperatures during much of the year, evaporative demand accelerates soil moisture depletion after wetting events. This produces rapid wetting–drying cycles and weaker autocorrelation in near-surface wetness, reducing the predictive value of lagged SSW. The district is characterized by intensive agriculture supported by canal irrigation and groundwater use (e.g., rice–wheat systems). Irrigation introduces abrupt, scheduled, and spatially heterogeneous wetting events that are not purely meteorologically driven. Such anthropogenic pulses reduce temporal smoothness and can create apparent “noise” in the GEOS-derived SSW signal, especially if irrigation processes are simplified or imperfectly represented in the underlying land-surface modeling. This makes the NASA POWER SSW series harder to emulate statistically using lags alone. GEOS-derived SSW is a spatially averaged product. In regions where land use, irrigation intensity, and soil properties vary at sub-grid scales, the gridded wetness series may reflect a mixture of processes and management practices. This can lower the signal-to-noise ratio and set an upper bound on attainable surrogate-model skill, even with sophisticated ML ensembles.

#### Model performance differences to climatic, topographic, and hydrological characteristics

Model performance differences across Moga, Hoshiarpur, and Firozpur can be linked to contrasts in climatic forcing, topography, and dominant hydrological controls on soil surface wetness (SSW). Hoshiarpur, being closer to the Shivalik foothills, generally experiences relatively higher and more reliable rainfall and stronger seasonal structure, which promotes smoother wetting–drying cycles and greater temporal persistence-conditions under which lag-based surrogate models perform better. Moga represents an intermediate central-plain setting with moderate rainfall and mixed rainfall–irrigation influence, yielding moderate persistence and correspondingly intermediate performance. In contrast, Firozpur shows weaker skill because its drier and more variable rainfall regime, higher evaporative demand, and strong dependence on canal/groundwater irrigation generate rapid, management-driven wetness fluctuations with reduced autocorrelation; combined with flat alluvial terrain and sub-grid heterogeneity, this increases noise in gridded GEOS-derived SSW and limits the predictive value of lag-only inputs. Uncertainty and robustness are important considerations when interpreting the performance of ensemble and hybrid models, particularly AdaBoost-based formulations. Boosting methods iteratively emphasize poorly predicted instances, which can improve bias but may also increase sensitivity to noise and weakly structured signals, raising the risk of overfitting. In the present study, this risk is evident in the divergence between training and testing performance in some scenarios, where improved training accuracy does not always translate into comparable gains during testing.

The robustness of the AdaBoost-hybrid models is therefore strongly conditioned by the statistical properties of the target SSW series. AdaBoost–LightGBM likely performs best because it combines the regime sensitivity of a tree structure with the smooth flexibility of logistic regression at the leaves, which is well suited to soil surface wetness (SSW) dynamics that exhibit both threshold-like shifts and gradual wetting–drying transitions. Compared with standalone RF, the AdaBoost wrapper iteratively reweights poorly predicted cases, improving fit during challenging periods such as seasonal transitions and wetness extremes and reducing systematic bias while retaining local interpretability. In contrast, tree-based and ensemble learners such as CART, RF, XGBoost, and LightGBM may still face difficulty in representing smooth wetness gradients when predictions are driven by partition-based structures, and their boosted variants can become more sensitive to noise when persistence is weak. Overall, AdaBoost–LightGBM offers a stronger bias–variance trade-off and a more process-consistent representation of regime-dependent SSW behaviour, explaining its superior relative performance within the lag-based surrogate modeling framework. In locations with stronger temporal persistence and seasonal structure (e.g., Hoshiarpur), boosting provides more stable generalization, whereas in locations characterized by rapid wetting–drying cycles and irrigation-driven variability (e.g., Firozpur), performance gains are modest and uncertainty remains high. The use of cross-validation, fixed parameter settings, and feature selection helped reduce overfitting; however, these measures cannot fully compensate for weak predictability inherent in the data.

These findings highlight that the superior performance of AdaBoost-based hybrids should be interpreted in a relative and conditional sense, rather than as universally robust. Explicit uncertainty characterization and incorporation of additional physical drivers are likely necessary to further improve robustness and reduce overfitting, especially in regions with highly variable soil moisture dynamics.

Because the analysis was conducted across only three selected sites, the findings should be interpreted as evidence from a limited-site methodological case study rather than as a basis for broad regional generalization. Accordingly, the present results are most useful for understanding the relative behavior of lag-based surrogate models under the examined site conditions, while broader generalization will require testing across a larger and more diverse set of locations.

## Limitations and applicability

The study area presents various limitations due to its diverse climatic variations across spatial and temporal scales. SSW in this area depends on several hydrological phenomena, as well as meteorological and geological settings. The proposed models are affected by the environmental and geological conditions specific to the region. Additionally, predicting, measuring, and using soil moisture data remains challenging due to the many factors influencing SSW, which can affect model reliability and applicability.

The primary limitation of this study arises from the nature of the soil surface wetness (SSW) dataset used for model development and evaluation. The SSW data obtained from the NASA prediction of worldwide energy resources (POWER) platform are not derived from direct in-situ soil moisture observations but are instead generated by the NASA Goddard Earth Observing System (GEOS) global circulation model coupled with land surface parameterizations. As a result, the machine learning models developed herein should be interpreted as surrogate (emulator) models designed to reproduce the temporal dynamics and statistical characteristics of GEOS-derived SSW estimates, rather than as predictors of true ground-measured soil surface wetness. This reliance on model-based reference data has important implications for real-world applicability. Any systematic bias, scale dependency, or structural uncertainty embedded within the GEOS modeling framework may be implicitly transferred to the surrogate models. Consequently, the proposed models are not intended to replace field-based soil moisture measurements or sensor networks for plot-level or farm-scale irrigation scheduling and operational decision-making. Caution should therefore be exercised when interpreting absolute SSW values, particularly in heterogeneous terrain or managed agricultural systems where local soil properties, land cover, and management practices exert strong controls on near-surface moisture dynamics.

An important limitation of the present study is that the NASA POWER/GEOS-derived SSW product is available at a relatively coarse spatial resolution (0.5° × 0.625°), which may not adequately represent local-scale heterogeneity in soil, topography, land use, and management conditions at the study sites. This coarse-grid representation introduces a scale mismatch between the model input/output data and site-level wetness dynamics, and such mismatch may partly explain the limited to moderate predictive skill observed in some cases. Accordingly, the present surrogate models should be interpreted as emulators of coarse-resolution GEOS-derived SSW dynamics rather than as tools for fine-scale field assessment or plot-level water management decisions.

The reliability of the results is most strongly influenced by three constraints. First, the reference SSW series is derived from NASA POWER (GEOS-based) modeled estimates, not in-situ observations; therefore, any structural bias, smoothing, or scale mismatch in the GEOS product may be inherited by the surrogate models, and the findings should be interpreted as emulation of POWER-consistent SSW rather than true ground wetness. Second, the models rely mainly on lagged SSW predictors, which capture persistence and seasonal memory but omit explicit physical drivers (e.g., precipitation, evapotranspiration, irrigation), potentially biasing performance toward locations with stronger temporal autocorrelation and reducing skill where wetness changes are rapid or management-driven (e.g., Firozpur). Third, AdaBoost-based hybrids can be sensitive to noise and may over fit in weak-signal settings, as indicated by training–testing performance gaps in some scenarios. Finally, the limited number of sites constrains regional generalization; results should be viewed as comparative and methodological, and broader spatial coverage and observational validation are needed to strengthen conclusions. Furthermore, the models are trained under historical conditions and assume stationarity in SSW dynamics; therefore, temporal extrapolation under future climate or land-use change, as well as spatial transfer beyond similar agro-climatic settings, may introduce additional uncertainty.

In addition, model performance during extreme wetness or drought events is inherently limited by the lag-based surrogate framework and the smoothing characteristics of the GEOS-derived SSW product. Extreme events are often short-lived and externally forced (e.g., intense rainfall or irrigation pulses), reducing temporal persistence and thereby limiting predictability from past SSW alone. As a result, model skill during extremes may be lower than that indicated by average performance metrics, and such periods should be interpreted with caution. While the framework is suitable for data-scarce environments due to its reliance on modeled inputs, the absence of ground observations increases uncertainty and limits validation, particularly in heterogeneous or intensively managed landscapes. Despite these limitations, the developed models remain relevant for several practical applications. The surrogate framework provides a computationally efficient alternative for generating NASA POWER–consistent SSW time series, which is particularly valuable for applications requiring rapid data generation, repeated simulations, or long-term historical analyses. Such applications include regional-scale planning, comparative scenario assessment, sensitivity analysis, and preliminary screening studies in data-scarce environments where long-term ground observations are unavailable. Moreover, by preserving the relative temporal variability and seasonal patterns of GEOS-derived SSW, the models can support decision-support systems and integrated modeling workflows that rely on consistent, spatially continuous wetness proxies rather than absolute field measurements.

Future research should focus on extending the present framework by incorporating ground-based soil moisture observations or satellite-derived products such as SMAP or ESA Climate Change Initiative (CCI) soil moisture datasets. Such integration would enable evaluation of the transferability of the surrogate models to true observational conditions and facilitate the development of hybrid frameworks that combine physically based modeling, remote sensing, and machine learning for improved soil moisture estimation across multiple spatial and temporal scales. The present findings also point to several promising directions for future research using hybrid and ensemble learning strategies. For example, the CART algorithm evaluated in this study can be naturally extended within Random Forest (RF) frameworks, where multiple CART trees are trained on bootstrapped samples with randomized feature selection to improve generalization and reduce variance. Such RF-based approaches may offer improved robustness compared to single-tree or simple bagging formulations, particularly in locations exhibiting high variability and noise.

Future studies may extend the present framework by examining hybrid ensemble architectures, stacked ensembles, or model-averaging strategies that combine CART, RF, XGBoost, LightGBM, LMT, and physically informed predictors. Integrating meteorological drivers (e.g., precipitation, temperature, radiation, wind speed) within these hybrid frameworks may help overcome the limitations of purely autoregressive inputs, especially in irrigation-dominated or semi-arid regions. Additionally, emerging approaches that combine ensemble learning with deep learning architectures (e.g., CNN–LSTM hybrids or attention-based temporal models) could be investigated to better capture nonlinear temporal dependencies and regime shifts in soil surface wetness dynamics. Such hybrid architectures would allow future work to balance physical interpretability, computational efficiency, and predictive performance, building upon the surrogate-modeling framework established in this study.

The proposed models should not be interpreted as direct tools for farm-level irrigation scheduling, since they emulate GEOS-derived SSW dynamics under a lag-based surrogate framework rather than field-observed soil moisture conditions. Accordingly, the practical value of the present approach lies more in methodological surrogate modeling and comparative assessment than in direct operational support for plot-scale irrigation decisions.

## Conclusions and future recommendation

This study shows that lag-based surrogate models can reproduce NASA POWER (GEOS-derived) soil surface wetness (SSW) with useful relative skill, but their performance depends strongly on the temporal structure of the target series. The models performed better where monthly SSW exhibited stronger persistence and seasonal continuity, indicating that antecedent wetness carried meaningful predictive information. In contrast, their performance was weaker where wetness dynamics were more irregular, rapidly changing, or influenced by local controls that were not represented by lagged SSW inputs alone. Accordingly, the main take-home message is that such lag-based surrogates are most suitable for methodological comparison, screening-level assessment, and relative emulation of modeled wetness dynamics, rather than precise farm-level decision-making or direct estimation of field moisture conditions. The results further emphasize that lag-based surrogate models are most applicable in regions where soil wetness exhibits clear seasonal structure and temporal memory, while their utility is limited in locations dominated by rapid, irrigation-driven or highly variable wetting–drying processes. Consequently, the proposed framework is best suited for screening-level analyses, comparative assessments, and decision-support applications that rely on relative wetness dynamics rather than precise estimation of absolute moisture values. Overall, this work contributes methodological guidance on when and where hybrid ensemble models can be reliably applied for soil wetness emulation using gridded model-based datasets. Future work should test these surrogate models against in-situ soil moisture observations, incorporate exogenous hydro-meteorological drivers, and evaluate whether their skill remains stable across more heterogeneous sites and management regimes. The following conclusions and recommendations can be drawn from the present study. The specific conclusions are outlined below.

1. The hybrid AdaBoost-based models showed better relative predictive performance than the bagging-based models for the study dataset, indicating their usefulness as surrogate tools for emulating GEOS-derived SSW dynamics under the tested conditions.
2. In the present study area, eight machine learning (ML) models were applied for the prediction of soil surface wetness (SSW). Among these, four were bagging-based models and four were hybrid models developed specifically for SSW prediction
3. Among the tested models, AdaBoost-LightGBM showed comparatively better agreement with the target SSW series at the selected sites. The relative advantage of AdaBoost-LightGBM was evident across the three study sites, although performance varied by location, indicating that model effectiveness remained site dependent.
4. For the AdaBoost-LightGBM model, CC values ranged from 0.61 to 0.76 in Moga, from 0.63 to 0.82 in Hoshiarpur (AdaBoost-LMT) and from 0.49 to 0.60 in Firozpur (AdaBoost-LMT). In terms of model error indices such as RMSE, it varies from 0.07 to 0.11 at Moga location, from 0.08 to 0.12 at Hoshiarpur location and 0.09 to 0.12 at Firozpur location. Similarly, nRMSE values ranged from 0.28 to 0.38 in Moga, from 0.25 to 0.27 in Hoshiarpur and from 0.31 to 0.32 in Firozpur during the testing phase.
5. The hybrid models show potential as screening-level surrogate tools for comparative wetness assessment, exploratory analysis, and broader environmental interpretation, but their present performance does not support direct field-scale operational use.
6. The use of high spatial resolution of satellite data and latest technologies enhances the assessment of SSW and improves the efficacy of hybrid models for the respective area.
7. Further improvements may be achieved by incorporating physically relevant predictors and uncertainty-aware modeling approaches, especially where SSW dynamics are governed by nonlinear or rapidly changing local conditions.
8. Future studies may evaluate surrogate model robustness under climate variability by integrating scenario analysis and longer-term environmental forcing data.
9. Further work is needed before these models can be used for crop-water requirement estimation, irrigation scheduling, or other precision management applications, particularly through validation against in-situ observations and incorporation of physically relevant predictors.
10. Coupling surrogate SSW models with physically based hydrological models may be explored in future research to combine computational efficiency with process-level interpretation.

Overall, the present findings should be interpreted as evidence on the conditions under which lag-based surrogate models can and cannot emulate modeled SSW, rather than as proof of direct applicability for farm-level water management decisions.

## Data Availability

The data will be made available online upon reasonable request to the corresponding author.
